# Rationally designed stapled peptides allosterically inhibit PTBP1–RNA-binding†

**DOI:** 10.1039/d3sc00985h

**Published:** 2023-07-13

**Authors:** Stefan Schmeing, Gulshan Amrahova, Katrin Bigler, Jen-Yao Chang, Joseph Openy, Sunit Pal, Laura Posada, Raphael Gasper, Peter 't Hart

**Affiliations:** a Chemical Genomics Centre of the Max Planck Society, Max Planck Institute of Molecular Physiology Otto-Hahn-Strasse 11 44227 Dortmund Germany peter.t-hart@mpi-dortmund.mpg.de; b Crystallography and Biophysics Unit, Max-Planck-Institute of Molecular Physiology Otto-Hahn-Strasse 11 44227 Dortmund Germany

## Abstract

The diverse role of the splicing factor PTBP1 in human cells has been widely studied and was found to be a driver for several diseases. PTBP1 binds RNA through its RNA-recognition motifs which lack obvious pockets for inhibition. A unique transient helix has been described to be part of its first RNA-recognition motif and to be important for RNA binding. In this study, we further confirmed the role of this helix and envisioned its dynamic nature as a unique opportunity to develop stapled peptide inhibitors of PTBP1. The peptides were found to be able to inhibit RNA binding *via* fluorescence polarization assays and directly occupy the helix binding site as observed by protein crystallography. These cell-permeable inhibitors were validated *in cellulo* to alter the regulation of alternative splicing events regulated by PTBP1. Our study demonstrates transient secondary structures of a protein can be mimicked by stapled peptides to inhibit allosteric mechanisms.

## Abstract 2

The dynamic role of a helix connecting the first two RNA-recognition motifs of PTBP1 was investigated for the design of an inhibitor. The developed stapled peptide interferes with RNA binding and was found to modulate an alternative splicing event regulated by PTBP1.

## Graphical abstract

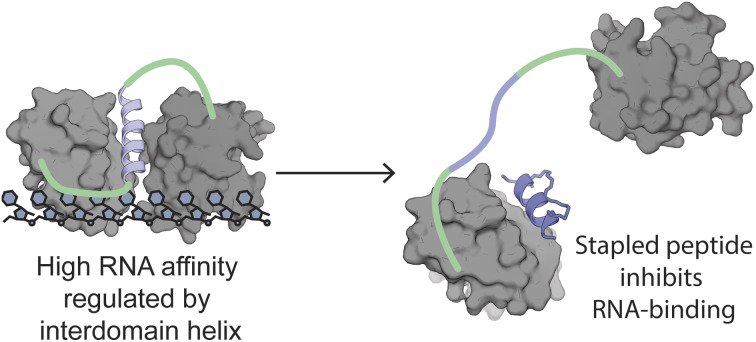

## Introduction

**Fig. 1:**
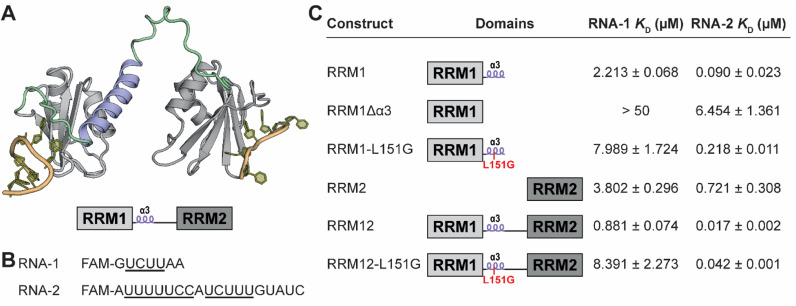
(A) Truncated PTBP1 AlphaFold structure of RRM1 and RRM2. The RNA binding sites were determined by overlaying NMR structures (PDB ID 2N3O and 2ADB) of the individual RNA bound RRMs over the truncated AlphaFold structure. Relative orientation of the RRMs towards each other is not accurate according to the model. The interdomain linker is indicated in green and the α3 helix in blue. A schematic representation is depicted below. (B) RNA sequences used in the FP assays. (C) Binding affinity of each PTBP1 variant for either RNA-1 or 2 as determined by fluorescence polarization.

Modulation of splicing has potential as a therapeutic strategy in various diseases including cancer, neurodegenerative diseases, viral infection, and cardiovascular disease.^1–9^ Although direct modulation of the spliceosome can be achieved by small molecule inhibition, this suffers from severe side effects arising from its essential function for splicing of nearly all transcribed mRNAs.^10^ Targeting splicing factors that regulate only a subset of splicing events is therefore an attractive alternative approach. One of such splicing factors is polypyrimidine tract-binding protein 1 (PTBP1) which regulates a distinct set of splicing events by binding to polypyrimidine rich sequences found in introns.^11–13^ It is part of the hnRNP family which typically have a repressive effect on splicing in the proximity of the sites they bind.^11,14,15^ Overexpression of PTBP1 is observed in various cancers, but it also plays a role in cardiovascular disease, viral infection, and neuronal development making it an attractive therapeutic target.^16–20^ PTBP1 binds RNA through four RNA-recognition motifs (RRMs), that together recognize the polypyrimidine sequence for high affinity binding.^13,21–23^ A canonical RRM is a small domain of approximately 90 amino acids and has a fold involving four β-strands and two α-helices in a β1α1β2β3α2β4 configuration.^23,24^ In the case of PTBP1 there are long flexible linkers in between RRM1 and 2 as well as RRM2 and 3 while RRM3 and 4 have a very short linker and are fixed in their position relative to one-another.^22^ A unique α3-helix is found in the linker between RRM1 and 2 (Fig. 1A), which can be considered to be part of RRM1. It stably folds upon RNA-binding and binds RRM1 itself, but in the absence of RNA the level of helix formation is reduced to only 17%.^25^ The unique structural feature was proposed to play a role in the RNA-binding of RRM1 itself but also in recognition of secondary structure elements in the bound RNA.^25,26^

Many RNA-binding proteins use one or more RRM domains for high affinity RNA-binding.^27^ Although they are involved in various aspects of RNA-biology, their role in the regulation of alternative splicing in cancer is especially attractive for therapeutic targeting.^1,2^ However, due to the lack of well-defined binding pockets on RRMs they can be considered “undruggable” and we therefore looked for alternative methods of inhibition.^28^ RRMs are often extended on either the N- or C-terminus with short motifs with a defined secondary structure similar to the PTBP1 RRM1 α3-helix.^27^ These extensions can be α-helical such the extra α-helix on the N-terminus of RRM1 of Dead End (DND1), the extra C-terminal α-helix on RRM3 of La, the extra α-helices both N- and C-terminal of the oRRM4 of Prp24, or the extra α-helix on the N-terminus of the RRM of *Saccharomyces cerevisiae* Snu17p.^29–32^ Besides α-helices other structural elements are also observed such as an αββ-extension on the N-terminus of RRM1 on Syncrip, the 3_10_-helix N-terminal of RRM2 in HuR, and the 3_10_-helix on the C-terminus of RRM2 of the *Drosophila melanogaster* Sex-lethal protein.^33–35^ We hypothesized that exploiting these unique structural features might provide an opportunity towards the design of inhibitors of RRM containing proteins in spite of the lack of pockets and maybe even provide a method to achieve selectivity.

Here, we first confirmed a role for the α3-helix in PTBP1–RNA-binding and exploited its dynamic nature for development of an inhibitor derived from this helix. By using a hydrocarbon peptide stapling approach, the helix could be mimicked to provide a peptide which can occupy its binding site and prevent PTBP1 from properly interacting with RNA. We demonstrate that such peptides bind to RRM1 and can indeed interfere with RNA-binding. After we confirmed them to be cell-permeable and have reasonable stability against cellular proteases we demonstrated they can modulate PTBP1 regulated RNA splicing events. We expect that this approach can become a general method for the design of RNA interaction inhibitors for RRMs and might also be applicable to other types of RNA-binding domains.

## Results

### Investigation of the role of the α3-helix in RNA-binding of PTBP1 RRM1 and 2

To confirm the role of the α3-helix described by the Allain group we performed fluorescence polarization (FP) experiments with various PTBP1 protein constructs and two RNA sequences.^25^ The first set of proteins contained either the single RRM1, RRM1 lacking the α3-helix (RRM1Δα3) or RRM1–L151G (Fig. 1A and C). The L151G mutation was previously described by the Allain group to break the fold of the helix and reduce RNA-binding 2.3 fold as measured by ITC.^25^ As tracer in the FP experiments, we used a FAM-labelled short RNA (RNA-1) with a single PTBP1 binding site (UCUU) which can accommodate a single RRM (Fig. 1B). The measured binding affinities for RNA-1 (Fig. 1C and S1†) indicated that the helix indeed contributes to the RNA affinity of RRM1 since its absence reduced the affinity strongly. The L151G mutation reduced binding 3.6-fold which is somewhat higher than what was observed previously but in the same range. Next, we measured the affinity of RRM2 and found that RRM1 more potently binds RNA-1, but only when the helix is intact. To see whether the effect of the helix was also present in larger constructs we measured the affinity of RRM1 and 2 combined (RRM12) as well as the RRM12–L151G mutant. The affinity of RRM12 is significantly enhanced over each individual domain, while the affinity of the L151G mutant for RNA-1 was strongly diminished to that of the single RRM1–L151G.

Next, we repeated the measurements with the longer RNA-2 which is the sequence of the microRNA miR-29b-2 and was previously reported to be recognized by PTBP1 (Fig. 1B, C, S1†).^36^ Since it has two poly(U) motifs, it is able to accommodate two RRMs and more accurately represents polypyrimidine tracts in mRNA. The affinities of all constructs improved for RNA-2 indicating an avidity effect. The overall trends stayed the same, where the affinity of RRM1 is better than RRM2 and is diminished when the helix is mutated or absent. For this RNA the affinity of RRM1–L151G was 2.4-fold lower than RRM1 which is identical to the reduction in affinity as observed by the Allain group. The double RRM constructs had a high affinity for RNA-2 in comparison to RNA-1 and the L151G mutation again reduced the affinity 2.5-fold. All used protein variants were analyzed by CD spectroscopy to verify folding and demonstrated identical spectral characteristics (Fig. S3C and D†).

The combined results indicated that the α3-helix indeed plays a role in RNA binding. Similar observations were made for other proteins that contain multiple RRM domains including Sex-lethal protein and DND1, where two RRMs orient their sheets to form a cleft for binding of neighboring RNA sites and where the interdomain linker folds into a helix.^29,35^

### Rationally designed stapled peptides inhibit PTBP1–RNA-binding

**Fig. 2:**
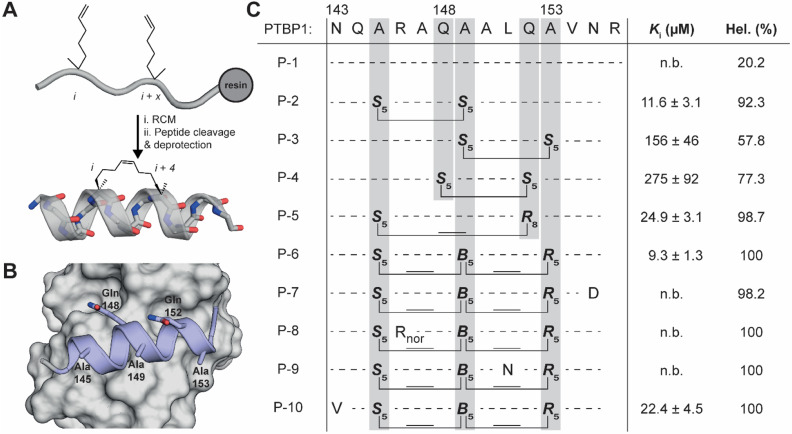
(A) General synthetic protocol for hydrocarbon stapling of an *i* to *i* + 4 staple. (B) PTBP1 RRM1 (grey) bound by the α3-helix (PDB 2N3O). Indicated are the amino acid positions used for hydrocarbon stapling. (C) Linear and stapled peptides derived from the PTBP1 α3-helix and their inhibitory potency in a competitive FP assay between RRM12 and RNA-2 (see Fig. 1). Also reported is the helicity as determined by CD spectroscopy.

Based on our results and those previously described on the role of the α3-helix, we hypothesized that an inhibitor bound to the RRM1 surface competing with the transient helix should disturb the RNA-binding capacity of RRM12. The α3-helix was used as a starting point to design helically stabilized peptides using hydrocarbon stapling. Hydrocarbon peptide stapling has been demonstrated to be a highly effective strategy to stabilize α-helical peptides and is facilitated by the introduction of amino acids bearing a terminal alkene during peptide synthesis (Fig. 2A).^37,38^ While the peptide is still on the solid-phase resin, these alkenes are cross-linked in a ring-closing metathesis reaction using a Grubbs catalyst. Different stapling strategies spanning single or multiple helical turns were reported, including *i*, *i* + 4 or *i*, *i* + 7 linkages.^37^ The stabilization and preorganization of the helical conformation leads to an increase in binding affinity due to a reduction in the entropic penalty upon binding to the target protein.^39^ Besides significant increases in affinity, hydrocarbon stapling can also significantly improve the cell permeability of the peptide making this a very attractive strategy to design inhibitors from α-helical template peptides.^40,41^ Hydrocarbon stapled peptides have been used to inhibit various PPIs, but to our knowledge have not yet been used to mimic transient helices dynamically binding to a domain in the same monomer. Furthermore, their use as inhibitors of protein–RNA interactions has not yet been explored widely with only a recent example demonstrating the inhibition between a pre-microRNA and its processing protein Dicer.^42^

For the initial design we used the *i*, *i* + 4 strategy where two (*S*)-pentenyl alanines (S_5_) were introduced in positions with three other amino acids in between. We picked three different amino acid pairs to replace with the S_5_ amino acids based on their orientation as observed in the NMR structures (P-2, P-3 and P-4, Fig. 2B and C).^25^ To prevent the staple from disturbing the interaction between the ligand and the protein we chose positions facing away from the binding site forming the pairs Ala145/Ala149, Gln148/Gln152, and Ala149/Ala153. The three peptides were synthesized on Rink amide resin and the linear peptides were treated with a Grubbs catalyst to connect the S_5_ residues. To avoid a mixture of *E*/*Z*-diastereomers we reduced the alkene in the linker of the staple using a solution phase hydrogenation protocol.^43^ The linear equivalent P-1 was synthesized for comparison but had to be extended on the C-terminus with an arginine residue to improve its solubility. The C-terminus is away from the binding surface and this modification was therefore not expected to have an impact on the binding affinity. The peptides were tested in a competitive fluorescence polarization assay using the RRM12 construct and RNA-2 (Fig. 2C and S2†). Linear peptide P-1 was not able to compete under the assay conditions, but the stapled peptides did to varying degrees. Peptide P-2 performed the best with a *K*_i_ value of 11.6 μM while the other peptides were less active at 156 and 275 μM (P-3 and P-4 respectively). As the staple is expected to stabilize the α-helical conformation we used circular dichroism (CD) spectroscopy to evaluate the helicity of the peptides. As expected, the most active peptide P-2 also demonstrated the highest helicity (Fig. 2C, S3A and B†), but the other two peptides did not follow this trend. It is possible that although P-4 was more helical than P-3, the replacement of Gln148 with the hydrophobic linker disturbed the interaction since the side chain amide potentially interacts with the RRM1 surface.

To further improve on P-2 we explored a second set of peptides by using different stapling strategies (Fig. 2C). For this we used the longer *i*, *i* + 7 staple, linking residues 145 and 152 (P-5), as well as the less commonly used *i*, *i* + 4, *i* + 7 linkage in a so-called stitched peptide linking residues 145, 149 and 153 (P-6).^44^ Since we did not observe the formation of *E*/*Z* isomers during the ring closing metathesis reaction for these peptides we avoided reduction of the double bonds to simplify the synthesis and improve yields. The new peptides were tested for activity in the competition assay and P-5 and P-6 demonstrated good activities of 24.9 μM and 9.3 μM respectively with higher helicities than P-2. We selected P-6 as our candidate for further studies and synthesized the scrambled control variant P-6S. When tested in the competitive FP assay, P-6S was not able to inhibit the interaction between RRM12 and RNA-2 (Fig. S2†).

### Mode of action of P-6

**Fig. 3:**
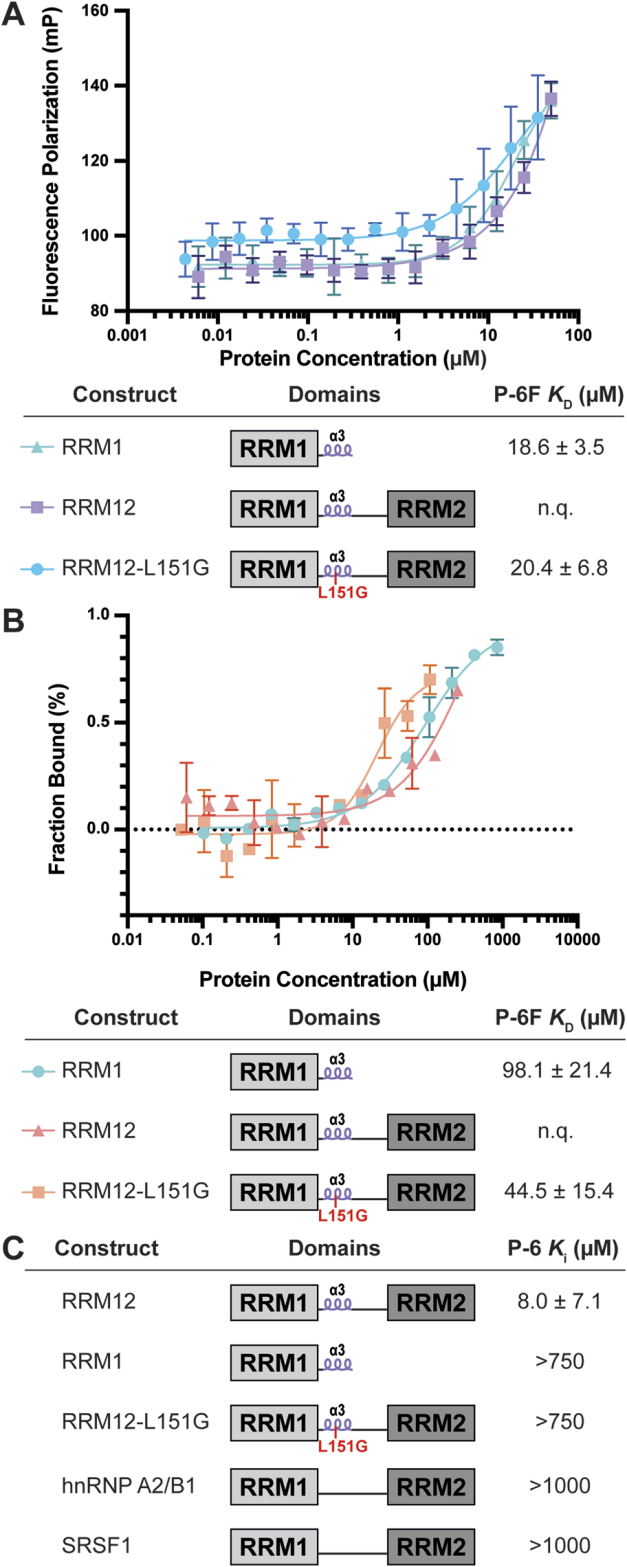
(A) Direct binding fluorescence polarization experiments of P-6F with indicated PTBP1 variants and measured *K*_D_ values. (B) Microscale thermophoresis analysis of P-6F with indicated PTBP1 variants and measured *K*_D_ values. (C) Inhibition of different constructs by P-6 evaluated using a competitive fluorescence polarization assay with RNA-2 (PTBP constructs) and RNA-AB and RNA-S for hnRNP A2/B1 and SRSF1 respectively. n.q. = not quantifiable.

Now that RNA–protein interaction inhibition was demonstrated, we set out to measure the affinity of P-6 for RRM1. To this end, we prepared the FITC-labeled variant P-6F and subjected it to a direct fluorescence polarization experiment by titrating it with either RRM1, RRM12, or RRM12–L151G (Fig. 3A). The titration demonstrated a *K*_D_ for RRM1 of 18.6 μM and a very similar affinity for RRM12–L151G (20.4 μM). Although an accurate *K*_D_ could not be determined for RRM12 due to the absence of saturation, the curve is shifted slightly indicating the affinity is only somewhat elevated. To confirm binding we used P-6F in a microscale thermophoresis experiment (Fig. 3B and S4†).^45^ The peptide was tested for binding to the same protein constructs as in the previous FP experiment. It demonstrated an affinity of 98.1 μM for the RRM1 which was higher than the *K*_i_ as measured in the competitive FP assay or the *K*_D_ from the direct binding FP experiment. Although binding was observed for RRM12 the curve was not complete, similar as in the direct FP experiment and an accurate *K*_D_ could therefore not be determined. However, the affinity improved with the RRM12–L151G construct to 44.5 μM.

Different PTBP1 variants were used to investigate the mode of action of P-6*via* competitive FP experiments. As illustrated in Fig. 1A there is a short linker between RRM1 and the α3-helix which also makes contacts with the RNA. It is possible that when P-6 occupies the binding site of the helix, this linker is displaced disrupting its interactions with the RNA and lowering the RNA-binding affinity. To test this, we measured whether P-6 could inhibit RRM1 RNA-binding. Interestingly, no inhibition was observed indicating that these interactions were not disrupted (Fig. 3C and S5†). Therefore, it seems most likely that P-6 inhibits RRM12 *via* disturbing the coordinated RNA-binding of the two RRM domains. As this is possibly regulated by the helix we tested if the peptide could inhibit RRM12–L151G where the role of the helix is reduced (Fig. 3C and S5†). Again, we observed that P-6 was not able to inhibit this variant and we hypothesized that since the helix doesn't play a role in this construct, occupying its binding site has no consequence. These findings further support a mode of action where P-6 disturbs coordinated RNA-binding by RRM1 and 2.

### Selectivity of P-6 for PTBP1

The RRM domain is the most commonly occurring RNA-binding domain in the proteome making it challenging to find selective inhibitors.^23,46^ Although various ligands have been described, their selectivity is rarely investigated, with an exception for the Musashi-1 inhibitor Ro 08-2750.^47–58^ To investigate whether P-6 has an effect on the RNA-binding of other dual RRM containing proteins, we evaluated its inhibitory potency against another hnRNP protein (hnRNP A2/B1) as well as a splicing factor from the SR-protein class (SRSF1).^1^ We evaluated P-6 in competitive fluorescence polarization assays using previously described RNAs recognized by these proteins.^59^ In both cases, no inhibition was observed up until 1 mM peptide concentrations (Fig. 3C and S6†).

### Structural evaluation of the RRM1Δα3:P-6 complex by X-ray crystallography

**Fig. 4:**
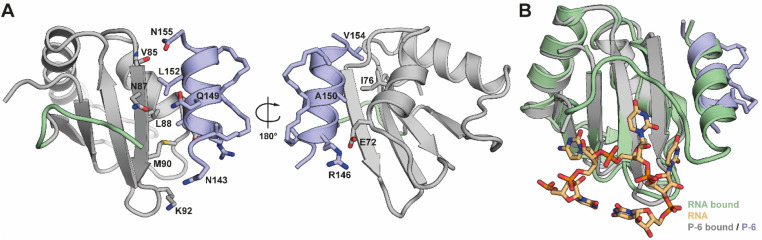
(A) Crystal structure of PTBP1 RRM1 (chain E) in complex with P-6 (chain e). Highlighted residues play a role in the PPI. PDB ID 8BWF. (B) Overlay of the RRM1:P-6 complex with the RNA bound structure of RRM1 (PDB ID 2N3O).

To validate the interaction of the stapled peptides with RRM1 we attempted co-crystallization of either peptide P-2 or P-6 with RRM1Δα3. We observed crystals with the stitched peptide (P-6) in space group *P*2_1_2_1_2 which diffracted to a resolution of 2.9 Å and had 16 dimers of protein–peptide complexes in the asymmetric unit (Fig. S7 and S8†). Generally, the structure (Fig. 4A) is on par with reported NMR structures of RRM1 in the RNA-bound (PDB 2N3O) and -unbound state (PDB 1SJQ) with RMSD-values of 1.14–1.56 and 1.48–1.73 Å respectively, after aligning all 16 protein chains (Tables S2 and S9†). This indicates that the domain more resembles the RNA-bound conformation upon binding of P-6. We could validate the binding mode of the peptide by helical electron density on the native binding site of the α3-helix in each of the dimers (Fig. S10A and S11†). Also, we observed density of the hydrocarbon linkers between the unnatural amino acids which, like expected from the design, point away from the binding site. The density of the staples validates our model and rules out any bias originating from using a structure containing the native helix during phasing of our model. The placement of P-6 overlaps well with the original α3-helix (Fig. 4B) and suggests the same hydrophobic binding core of I76, L80, V85, M90, and L88 on the RRM1 domain and A150, L152 and V154 on P-6 (Fig. 4A). Additionally, polar interactions between E72 (RRM1) and R146 (P-6), the carbonyl oxygen of V85 (RRM1) and N155 (P-6), and side-chain–side-chain interactions of N87 (RRM1) and Q148 (P-6) are observed. No significant conformational changes were observed in RRM1 upon binding of our stapled peptides in comparison to the RNA-bound state (Fig. S10B†). However, the influence of P-6 competing with the native helix can't be observed with this protein structure, as the native α3-helix is not part of the protein construct used in this experiment. Since the peptide does not obscure the RNA-binding site of RRM1 (Fig. 4B) we hypothesize that the stapled peptides block the correct orientation of both RRMs for high RNA affinity.

### Point mutation of P-6

After obtaining the crystal structure of P-6 bound to RRM1 we attempted to optimize the affinity of the peptides by introducing mutations *via* rational design (Fig. 2C). We designed four mutants where we hypothesized it was possible to make new or more optimal interactions. By mutation of the terminal Asn155 to Asp we hoped to form a new electrostatic interaction with RRM1 Lys84 (P-7). P-6 residue Arg146 forms an electrostatic interaction with RRM1 Glu72 but does so in an extended fashion. By shortening the Arg side chain to nor-arginine (P-8) we hoped to reduce the entropic penalty paid when the flexible arginine side chain is restricted this way. The side chain of P-6 residue Leu151 seems to bind a pocket lined with various polar groups such as backbone amides. To introduce the possibility for new hydrogen bonds we mutated it to Asn (P-9). In the opposite way, P-6 residue Asn143 binds a rather hydrophobic pocket, and we therefore mutated it to Val (P-10). Unfortunately, none of the peptides demonstrated improved affinity suggesting a larger variety of modifications needs to be explored to identify higher affinity peptides.

### Stapled peptides regulate the splicing of PTBP1 targets *in cellulo*

**Fig. 5:**
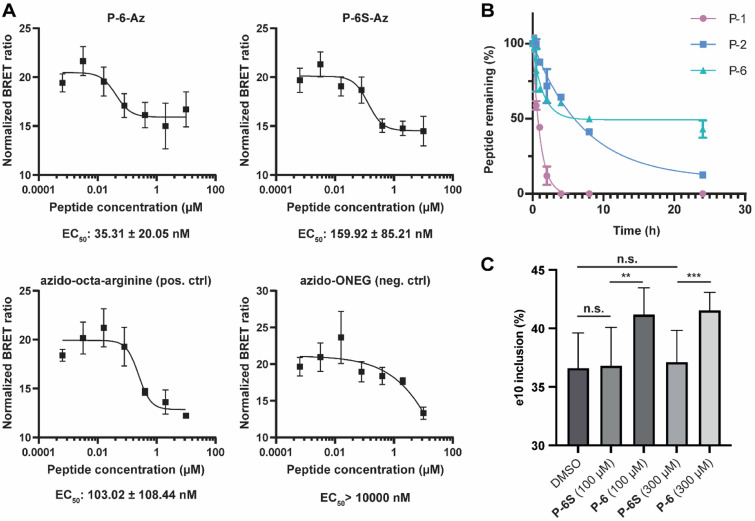
(A) Nanoclick assay results in HEK293T cells treated with azide-labeled peptides for 20 hours. (B) Evaluation of the stability of P-1, P-2, and P-6, in HEK293T cell lysate by HPLC. (C) RT-PCR analysis of PTBP2 exon 10 inclusion in HEK293T cells after treatment with peptides P-6 and P-6S at 100 and 300 μM. **: *p* = 0.0056, ***: *p* = 0.0008; Student's *t*-test.

Stapled peptides have been reported to be able to pass cell membranes with high efficiency, but their cell permeability is dependent on the peptide sequence and the position of the staple.^41^ To evaluate whether the α3-helix derived peptide is cell permeable, we used azidolysine derivatized variants of P-6 and P-6S (P-6-Az and P-6S-Az) and used them in the NanoClick assay.^60^ In this assay cells are transfected to express a NanoLuc–HaloTag fusion protein followed by treatment with a dibenzoazacyclooctyne reagent modified with a chloroalkane (DIBAC–CA) that covalently modifies the HaloTag protein. When an azide modified NanoBRET acceptor (NB618-Az) is added, it will undergo strain promoted azide–alkyne cycloaddition (SPAAC) with the DIBAC–CA placing the acceptor in the proximity of the NanoLuc protein leading to an observable BRET signal. When the cells are first treated with an azide containing peptide, it can occupy the DIBAC position and inhibit the BRET signal. The SPAAC reaction only happens if the peptide is able to reach the cytosol and the assay therefore provides a read-out of cell-permeability. The cells were incubated with the peptides for 20 hours and as controls, we used peptides described in the report on the development of the assay which are azido-octa-arginine as positive control and azido-ONEG as negative control.^60,61^ After background correction (raw data in Fig. S14†), the EC_50_ values for the control peptides were very similar to the reported values (azido-octa-arginine: 89 ± 12 nM, and azido-ONEG > 10 000 nM) and a >100-fold difference also in line with the original report.^60^ The results show that P-6-Az has very good cell permeability with an EC_50_ value of 35 nM significantly surpassing that of the positive control peptide (Fig. 5A). The high uptake is similar as for the stapled peptide MP-081 which was previously found to have an EC_50_ value of 48 ± 7 nM in the same assay.^60^ The scrambled P-6S-Az also has good membrane permeability and is comparable to the positive control. Next, we investigated the stability of P-6 in cell lysate in comparison with the monocyclic P-2 and linear P-1 (Fig. 5B). The linear P-1 was fully digested after 4 hours, while the single stapled P-2 was still 12% intact after 24 hours. As expected, the stitched P-6 was significantly more stable with 43% still intact after 24 hours. Although a significant amount of peptide was degraded, we deemed the stability to be sufficient for cellular experiments. To this end, we set out to evaluate whether they were able to interfere with the function of PTBP1 in the alternative splicing regulation of exon 10 of PTBP2.^20,62^ First, we validated the PTBP1 dependent regulation of exon inclusion by knockdown of PTBP1 in HEK293T cells and observed increased inclusion of PTBP2 exon 10 (Fig. S12B and C†). Cells were then treated with peptides at 100 and 300 μM and the results (Fig. 5C and S12A†) demonstrated that P-6 significantly increased the inclusion of exon 10 of PTBP2 in comparison to P-6S. Cell viability was not affected by P-6 at concentrations of up to 300 μM (Fig. S13†). Since both concentrations led to the same level of inclusion, it seems that the effect is already saturated at 100 μM which corresponds to the observed *K*_i_ values and good cellular uptake of P-6.

## Conclusions

**Fig. 6:**
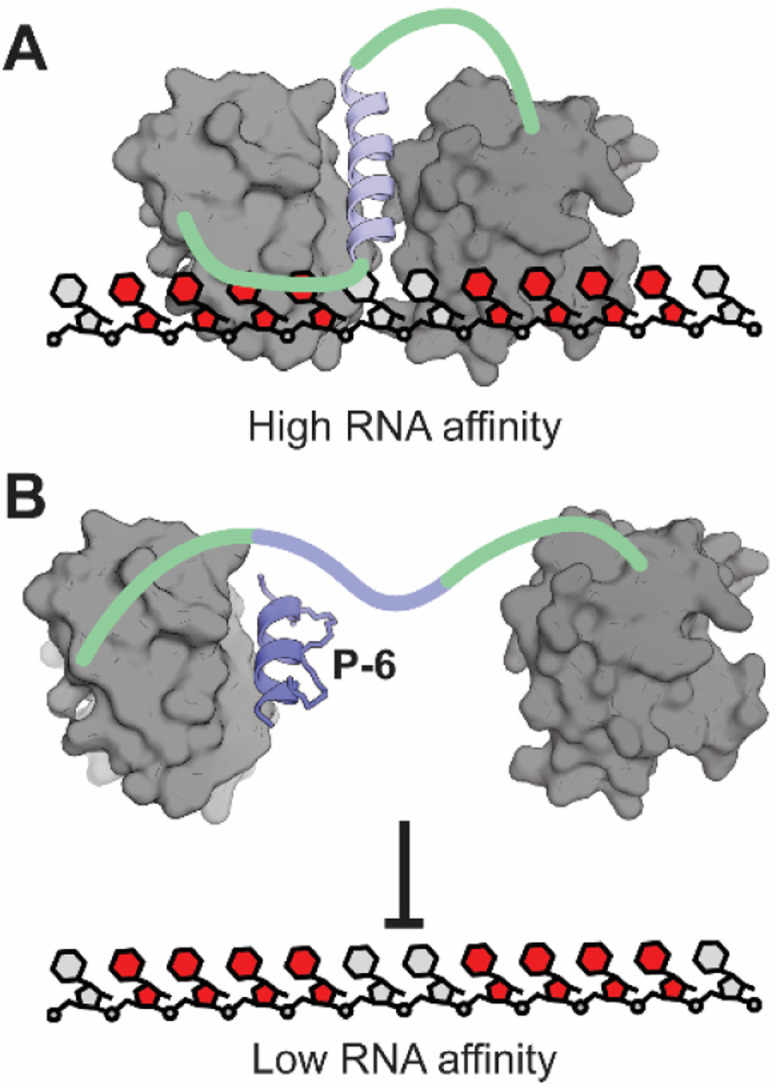
(A) When the α3-helix is able to fold and bind RRM1, the RRM12 construct has a high affinity for RNA. (B) If the α3-helix binding site is occupied, the affinity for RNA is decreased.

In this study we confirmed a previously proposed role of a transient helix interacting with PTBP1 RRM1 during RNA-binding.^25^ We confirmed that the affinity of RRM1 for poly(U) RNA is increased by the extra α3-helix and speculated that it needs to be formed to orient both RRMs properly for high affinity RNA-binding. Our findings are in line with recent structural studies from the Allain group indicating the formation of the transient helix after RNA-binding of RRM1 by integrative structural biology approaches.^25,26^ We hypothesized that occupying the binding site of the transient helix would be a suitable strategy for inhibiting the interaction between PTBP1 and RNA (Fig. 6). To this end, we synthesized a set of hydrocarbon stapled peptides that mimic the helix and identified P-6 as a potent inhibitor of RNA-binding with a *K*_i_ of 9.3 μM. When tested against several different protein constructs, P-6 did not show any inhibitory effects on the individual RRM1 or on the RRM12 construct with a L151G mutation which disturbs the role of the helix. The fact that RNA-binding can only be inhibited efficiently in the presence of both RRMs and an intact helix confirms that there is a role for the α3-helix in RNA-binding. Direct binding FP and microscale thermophoresis experiments confirmed that P-6 binds to RRM1, which could be confirmed further *via* protein crystallography. The co-crystal structure clearly demonstrates that the peptide occupies the α3-helix binding site on RRM1 as the design intended. The NanoClick assay showed that both P-6 and its scrambled equivalent P-6S were able to reach the cytosol with high efficiency. Stability assays testing the integrity of the peptides after exposure to cellular proteases in cell lysate demonstrated that especially P-6 was stable over longer times and had superior stability in comparison with the monocyclic P-2 or linear P-1. RT-PCR experiments demonstrated that P-6 was able to modulate the well-studied PTBP1 regulated splicing event of inclusion of exon 10 in PTBP2. These findings provide further evidence that not all four RRM domains play a role in each splicing event that PTBP1 regulates. Such domain specific splicing regulation of PTBP1 has previously been described to be influenced by protein binding partners that bind either RRM1 (MCL1) or RRM2 (Raver1).^63,64^ The described inhibitors of PTBP1 are first-in-class and act through an unprecedented mode of action for RNA-binding inhibition. Although further studies on selectivity are necessary, the lack of activity on two alternative dual RRM proteins is promising. By demonstrating that intramolecular interactions can be disrupted to inhibit RNA binding we provide a novel way to target RNA-binding proteins that are otherwise devoid of well-defined pockets and could be considered “undruggable”.^28^

## Data availability

Crystallographic data for the PTBP1 RRM1Δα3:P-6 complex has been deposited at the Protein Data Bank under accession number 8BWF and can be obtained from https://www.rcsb.org/structure/8BWF.

## Author contributions

PtH conceived the project. SS and KB performed biophysical experiments with PTBP1 constructs. GA, JYC, JO, and SP synthesized stapled peptides. SS performed direct binding FP, competitive FP, and MST experiments with PTBP1 constructs. GA performed competitive FP experiments with hnRNP A2/B1 and SRSF1 constructs. SS and RG performed crystallographic analysis of the RRM1Δα3:P-6 complex. SS and LP performed NanoClick experiments. GA performed cell lysate stability experiments. SS and KB performed PTBP1 knockdown and splicing experiments. PtH supervised the experiments and acquired funding for the project. PtH and SS performed data analysis and wrote the manuscript. All authors have given approval to the final version of the manuscript.

## Conflicts of interest

There are no conflicts to declare.

## Supplementary Material

SC-014-D3SC00985H-s001
