# Supplementary material for: Rationally designed stapled peptides allosterically inhibit PTBP1–RNA-binding
Source: Chem Sci. 2023 Jul 13;14(31):8269–78. doi: 10.1039/d3sc00985h (PMC10411625; doi:10.1039/d3sc00985h)
Supplement: SC-014-D3SC00985H-s001 [file SC-014-D3SC00985H-s001.pdf]

Electronic Supplementary Material (ESI) for Chemical Science.
This journal is © The Royal Society of Chemistry 2023

# Supporting Information

## Rationally designed stapled peptides allosterically inhibit PTBP1 RNA-binding

Stefan Schmeing,[a] Gulshan Amrahova,[a] Katrin Bigler,[a,b] Jen-Yao Chang,[a] Joseph Openy,[a] Sunit Pal,[a] Laura Posada,[a] Raphael Gasper,[c] Peter 't Hart[a,*]

[a] Chemical Genomics Centre of the Max Planck Society, Max Planck Institute of Molecular Physiology, Otto-Hahn-Strasse 11, 44227, Dortmund, Germany

Email: peter.t-hart@mpi-dortmund.mpg.de

[b] Current Address: Faculty of Chemistry and Chemical Biology, TU of Dortmund University, Dortmund, 44227, Germany

[c] Crystallography and Biophysics Facility, Max Planck Institute of Molecular Physiology, Otto-Hahn-Strasse 11, 44227, Dortmund, Germany

# Contents

1. Biological methods ..... 3
2. Synthetic methods ..... 9
   Reagents ..... 9
   Synthesis of Peptides ..... 9
      Linear Peptide Synthesis ..... 9
      Acetylation ..... 10
      FITC Labelling ..... 10
      Ruthenium Olefin Metathesis ..... 10
      Peptide cleavage ..... 10
      Stapled Peptide Reduction ..... 10
      Synthesis of azido-octa-arginine and azido-ONEG ..... 11
      P-1 ..... 13
      P-2 ..... 14
      P-3 ..... 15
      P-4 ..... 16
      P-5 ..... 17
      P-6 ..... 18
      P-7 ..... 19
      P-8 ..... 20
      P-9 ..... 21
      P-10 ..... 22
      P-6-Az: ..... 23
      P-6S-Az: ..... 24
      P-6S: ..... 25
      P-6F: ..... 26
      DIBAC-CA: ..... 27
      azido-ONEG: ..... 28
      Azido-octa-arginine: ..... 29
3. Figures ..... 30
4. Tables ..... 38
5. Biologicals ..... 40
   Used Oligonucleotides, Antibodies and siRNAs ..... 40
      References ..... 41

# 1. Biological methods

**Bacterial Methods**

*E. coli* BL21 DE3 RIL (Invitrogen) was used for recombinant expression of proteins. Cloning and plasmid amplification was either done in *E. coli* Top 10 F' (Invitrogen) or DH10B (Invitrogen). Transformations were performed using heat shock protocols and plated on LB-Agar plates with ampicillin (100 µg/ml).

**Cloning**

Constructs with RRM1, RRM2 and RRM12 were cloned into linearized pMAL vector (NEB) using the restriction enzymes BamHI and XhoI. The RRM1Δα3 construct for crystallography was subcloned into pOPIN-His-MBP using KpnI and HindIII restrictions sites. Inserts were generated by PCR using Phusion polymerase (NEB) from a cDNA clone (Dharmacon; GenBank: BC013694.1) and inserted via SLIC or CPEC.[1,2] Individual clones were verified by Sanger sequencing after MiniPrep (ThermoFisher).

The L151G mutation in RRM1 and RRM12 was generated by site directed mutagenesis using complementary oligonucleotides. Two separate reactions with either forward or reverse primer cycled 3 times followed by 15 cycles after pooling both reactions. This reaction was DpnI digested (ThermoFisher) and plated on selective LB-Agar plates after transformation into *E. coli* DH10B or Top10F'. Clones were verified by Sanger sequencing after MiniPrep (ThermoFisher).

**Purification of PTBP1 variants**

In this study the following protein constructs were used: MBP-RRM1 (G41-A163), MBP-RRM2 (M177-D284), MBP-RRM1-L151G (G41-A163, mutation L151G) and RRM12 (P57-V265), RRM12-L151G, MBP-RRM1Δα3(G41-S140).

All proteins were purified using the following protocol: *E. coli* BL21 DE3 RIL were transformed with plasmids encoding for the protein of interest by heat shock. Afterwards, a pre-culture in LB medium (100 µg/ml Ampicillin) was inoculated with a single, fresh colony and grown over night (37 °C, 180 rpm). An expression culture was inoculated 1:200 with the pre-culture and cultivated at 37°C, 180 rpm until an $OD_{600}$ of ca. 0.6 was reached. After chilling (4 °C, 30 min) protein expression was induced with 200 µM IPTG overnight at 20 °C, 180 rpm. Cultures were harvested at 5000 x *g* and the pellets resuspended in Buffer A with 0.1 mM PMSF (50 mM Tris pH 8.0, 200 mM NaCl). After addition of a spatula tip of lysozyme and DNAse I (Sigma Aldrich) the suspension was incubated at 4 °C for 30 min while stirring. Following lysis by sonification the solution was cleared by centrifugation (60000 x *g*, 1 h, 4 °C) followed by filtration through a 0.22 µm filter. All FPLC based methods were performed using an ÄKTA Explorer FPLC system (GE Healthcare). The protein was first purified by nickel affinity chromatography (His-Trap 5 ml, GE Healthcare) using a gradient of Buffer B (50 mM Tris pH 8.0, 200 mM NaCl, 500 mM Imidazole) followed, if needed, by tag-cleavage during dialysis against Buffer A with His-tagged TEV-protease or His-tagged 3C-protease. Non-cleaved protein and protease were removed by collecting the unbound fraction of a Ni-NTA column. Afterwards, the solution was diluted in 50 mM Tris pH 8.0 to reach a NaCl concentration of <=50 mM and separated on a Heparin column (Heparin HP 5ml, GE Healthcare) using a gradient of high salt buffer (50 mM Tris pH 8.0, 2 M NaCl). This was followed by

size exclusion chromatography (Superdex 75 16/60 or 26/60) with SEC buffer (50 mM Tris, 200 mM NaCl). Protein homogeneity was assessed by the size exclusion chromatogram and purity was checked by SDS-PAGE analysis to be >= 90%.

**Purification of SRSF1**

The gene encoding for SRSF1-RRM12 (1-195) was subcloned into pOPIN-His expression vector and expressed in *E. coli* BL21 (DE3). A pre-culture of transformed bacteria was made followed by expression in LB-Medium (100 µg/ml Ampicillin, 1 mM IPTG, 18 °C, 160 rpm overnight) after growing the culture at 37 °C, 160 rpm to an $OD_{600}$ of 0.6. The harvested cells (5000 x *g*, 4 °C, 15 min) were resuspended in Buffer $A_{SRSF1}$ (50 mM $Na_2HPO_4$ pH 8.0, 300 mM KCl, 50 mM L-Arg, 50 mM L-Gly, 1.5 mM $MgCl_2$) supplemented with 1 mM PMSF followed by lysis through sonification. The protein was purified using a HisTrap HP 5 ml column (GE-Healthcare) using a gradient of Buffer $B_{SRSF1}$ (50 mM $Na_2HPO_4$ pH 8.0, 300 mM KCl, 50 mM L-Arg, 50 mM L-Gly, 1.5 mM $MgCl_2$, 500 mM Imidazole) followed by dialysis into wash buffer (50 mM $Na_2HPO_4$ pH 8.0, 300 mM KCl, 50 mM L-Arg, 50 mM L-Gly, 1.5 mM $MgCl_2$, 40 mM Imidazole) and a second run of the affinity column. The protein containing fractions were afterwards dialyzed into wash buffer with tag-cleavage using His-tagged 3C protease. The cleaved construct was subsequently used for a third affinity column run collecting the unbound fraction which was dialyzed into storage buffer (20 mM $Na_2HPO_4$ pH 8.0, 150 mM KCl, 50 mM L-Arg, 50 mM L-Gly, 1.5 mM $MgCl_2$, 0.2 mM EDTA, 1 mM TCEP).

**Purification of hnRNP A2/B1**

The MBP-tagged hnRNP A2/B1 construct (1-251) was expressed from a pOPIN-His-MBP vector in *E. coli* BL21 (DE3) RIPL cells. A pre-culture in TB (0.01% lactose, 2mM $MgSO_4$, 100 µg/ml ampicillin, 50 µg/ml chloramphenicol) was used to inoculate the expression culture with a starting $OD_{600}$ of 0.05 at 37 °C. Expression was autoinduced at 37 °C for 4 h followed by expression over night at 25 °C. Harvested cells (5000 x *g*, 15 min, 4 °C) were resuspended in Buffer $A_{hnRNPA2/B1}$ (50 mM HEPES pH 8.0, 300 mM NACl, 20 mM Imidazole, 1 mM TCEP) supplemented with 1 mM PMSF) and lysed with a microfluidizer. Immobilized metal affinity chromatography was performed using a HisTrap FF crude 5 ml column. The loaded protein was washed with wash buffer (50 mM HEPES pH 8.0, 300 mM NaCl, 30 mM Imidazole, 1 mM TCEP) and eluted using elution buffer (50 mM HEPES pH 8.0, 300 mM NaCl, 500 mM Imidazole, 1 mM TCEP). The protein containing fractions were further purified with size exclusion chromatography (Superdex 75 26/60 prep grade column) into storage buffer (50 mM HEPES pH 8.0, 100 mM NaCl, 1 mM TCEP).

**Fluorescence polarization assay with PTBP1**

The buffer of the purified proteins was exchanged using spin columns into FP $buffer_{PTBP1}$ (20 mM Sodium phosphate buffer pH 8.0, 50 mM NaCl, 0.001% Tween20). For binding assays of protein and RNA the protein was serially diluted 1:1 in FP buffer. Fluorescently FAM labeled RNA was added to a final concentration of 5 nM and a final reaction volume of 20 µl. After equilibrating (20 °C., 30 min) the fluorescence polarization was measured in a plate reader (TECAN Spark, monochromators: Exc. 490 ± 10 nm; Em. 520 ± 10 nm). Competitive assays were performed using a final RNA concentration of 5

nM and a final protein concentration that corresponded to 50-70 % binding of the probe. Competitors were dissolved in FP buffer and 1:1 serially diluted before addition of pre-incubated protein-RNA complex, followed by equilibration (20 °C, 30 min) and readout as mentioned before. Experiments were performed in triplicates if not otherwise mentioned to be duplicates.

Fluorescence polarization assays with purified PTBP1 constructs and FITC-labeled peptides were performed in FP $buffer_{PTBP1.}$ The protein buffers were exchanged into FP buffer using spin columns and serially diluted in the same buffer. Afterwards, 20 nM fluorescently labeled **P-6-F2** was added to reach a final concentration of 10 nM probe. Fluorescence polarization was read out in a TECAN Spark with the settings mentioned previously.

### Fluorescence polarization assay with SRSF1

Purified proteins were buffer exchanged into FP-$buffer_{SRSF1}$ (20 mM $Na_2HPO_4$ pH 7.0, 150 mM KCl, 1.5 mM MgCl2, 0.2 mM EDTA, 50 mM L-Glu, 50 mM L-Arg, 1 mM TCEP, 0.01 % Triton X-100) using spin columns. Competitive assays were set up using 1 nM Cy5-labeled RNA-S and 400 nM SRSF1 which were added to serially diluted competitors dissolved in FP-$buffer_{SRSF1}$. Fluorescence polarization was measured in a plate reader (TECAN Spark; filters: Exc. 610 ± 20 nm; Em. 670 ± 25 nm).

### Fluorescence polarization assay with hnRNPA2/B1

Purified hnRNPA2/B1 was buffer exchanged into FP-$buffer_{hnRNPA2/B1}$ (25 mM HEPES pH 8.0, 100 mM NaCl, 1 mM TCEP, 0.01 % Triton X-100) using spin columns. For the competitive assay 10 nM hnRNPA2/B1 and 1 nM of Cy5-labeled RNA-AB were added to serially diluted competitors in FP-$buffer_{hnRNPA2/B1.}$ Fluorescence polarization was measured in a plate reader (TECAN Spark; filters: Exc. 610 ± 20 nm; Em. 670 ± 25 nm).

### Microscale thermophoresis

The different protein constructs were buffer exchanged into FP buffer using spin columns and afterwards serially diluted into FP buffer. Peptide P-6F was dissolved in FP buffer containing 0.04 % Tween 20. P-6F was added to the serially diluted protein to a final concentration of 100 nM and the solutions were loaded into Monolith NT.115 premium capillaries and subsequently measured with the blue light source and high MST-Power with a Monolith NT.115 instrument. The data was evaluated at time points with best signal to noise ratio and evaluated using the manufacturers software. All experiments were performed in duplicates.

### Circular Dichroism Spectroscopy

Circular dichroism was measured using 50 µM peptide solutions in CD buffer (20 mM sodium phosphate buffer pH 8.0, 10 mM NaF) in triplicates at 20 °C with a JASCO J-815 CD spectrometer and 1 mm pathlength. Secondary structure composition of peptides was calculated using JASCO multivariate secondary structure analysis with a reference data set. For experiments with PTBP1 constructs the

protein was buffer exchanged into CD-Buffer using spin columns and measurements were performed in this buffer under similar conditions with protein concentrations of 0.2 mg/ml.

**Co-Crystallisation of RRM1Δα3 with P-6**

Protein construct RRM1Δα3 was purified using the protocol discussed above and concentrated to 14 mg/ml. **P-6** was dissolved in SEC buffer and added in a 1.5 molar excess to the protein solution. After removal of particles by centrifugation (20000 x *g,* 4 °C, 10 min) sitting-drop experiments were set up by adding 200 nl of protein/ligand to 100 nl reservoir solution (1.89 M $(NH_4)_2SO_4$, 0.1 M HEPES pH 6.86, 2% v/v PEG400) in MRC 3-drop plates. Sealed plates were incubated at 20 °C using a Formulatrix RockImager and crystals were obtained after several days. Prior to flash-freezing the crystals were cryo conserved by adding 0.5 µl reservoir solution containing 20% v/v glycerol to the drop.

**X-ray data collection and processing**

X-ray diffraction data was collected at X10SA (PXII) at Swiss Light Source. Datasets were integrated using the XDS package and scaled using XSCALE.[3] The structure was solved using phaser (Phenix) and a truncated Alphafold model of RRM1 with the helical peptide present. The crystal had a high disorder according to the weak density of several dimers. When comparing the 16 RRM monomers to each other, they showed a reasonable similarity (RMSDs 0.45 – 0.89 Å) and most differences were observed in the unstructured loops. Some chains show a significantly higher disorder compared to others that are indicated by substantially higher B-factors and lower map quality. The electron density map further indicates an alternative conformation of a potential domain swap between two chains at residues 118 – 127 at low occupancy. This is why we have decided to only build the model without domain swap.

**Structure solution and refinement**

RRM1Δα3•**P-6** crystallized in $P\ 2_1\ 2_1\ 2$ spacegroup with dimensions 244.37 x 76.83 x 94.19 Å with 32 molecules in the asymmetric unit (16 dimers of protein bound to peptide). The structure was solved using a truncated model of human PTBP1 RRM1 from alphafold with Phaser (Phenix Suite).[4,5] The resulting model was refined in iterations of phenix.refine and manual model building in Coot.[5,6] The structure was refined to a final $R_{free}$ of 34% at 2.9 Å.

**Cell culture**

HEK293T cells (DSMZ) were maintained in DMEM supplemented with 10% FBS with 1x Pen-Strep (Gibco) at 37 °C, 5% $CO_2$.

**Viability Assay**

HEK293T cells were seeded at 30000 cells/well in 75 µl DMEM supplemented with 10% FBS with 1x Pen-Strep (Gibco) in 96-well plates on the day prior the experiment. After adhering the cells were treated with a dilution series of peptides in DMSO (final DMSO concentration 0.5%). Cell viability was read out using CellTiterGlo 2.0 (Promega) according to the manufacturers protocol. Viability was calculated in % of signal normalized to the DMSO controls.

**RT assays**

HEK293T cells were seeded with a density of 20000 cells/well in 96-well plates and cells adhered for 24 h. Afterwards, the cells were treated with peptides **P-6**, **P-6S** or DMSO to reach final concentrations of 300 µM, 100 µM and final DMSO concentrations of 0.5 %. After 24 h of treatment, total RNA was isolated using a Qiagen RNeasy Mini Kit with DNase on-column digestion. The RNA was eluted in 30 µl nuclease free water and was directly further purified and concentrated by ethanol precipitation to increase the RNA quality and concentration. The RNA was precipitated by adding 0.1 volume 3M NaAc, 3 volumes ice cold ethanol and GlycoBlue™ co-precipitant (ThermoFisher Scientific). After chilling at -20°C for 30 min the RNA was pelleted by centrifugation in a table-top centrifuge (30 min, 4 °C, 14000 x *g*). The pellet was washed twice with 500 µl ice cold 70 % ethanol followed by centrifugation (10 min, 4 °C, 14000 x *g*). After removal of supernatant the pellet was air-dryed in an RNase free environment and the pellet resolubilized using 10 µl nuclease free water. Reverse transcription reactions were performed using 500 ng of RNA and High-Capacity cDNA reverse transcription kit (ThermoFisher Scientific) following the manufacturer's instructions. From these reactions 1 µl of cDNA was used to amplify PTBP2 exon10 with Hot Start *Taq* DNA polymerase (NEB) following the manufacturer's instructions (45 cycles, 60 °C annealing temperature). Those PCRs were analyzed using a 2 % agarose gel (1x TAE) and band intensities were determined using BioRad Image Lab.

**Knockdown with siRNA**

For knockdowns 30000 cells/well HEK293T cells were seeded in 6 well plates. After adhering overnight, knockdowns with PTBP1-siRNA (Dharmacon) or control siRNA (Dharmacon) were performed with Lipofectamine RNAimax (ThermoFisher) according to the manufacturer's instructions. Cells were cultivated as stated above for 48 h and lysates were prepared for Western blotting.

**Western Blotting**

After culturing of cells in 6-well plates, the cultures were washed with ice cold PBS twice. Then, 200 µl NP40-Buffer (50 mM Tris pH 8.0, 150 mM NaCl, 1.0% NP-40) supplemented with 1X Complete protease inhibitor cocktail (Takara Bio) were added to the cells and incubated for 20 min at 4 °C on a rocking table. The mixture was resuspended and added to microcentrifuge tubes and further incubated on a rotary shaker at 4 °C for 30 min. Lysis was completed by rigorous resuspension with a P20 pipette, and the debris was removed by centrifugation (20000 x *g*, 4 °C, 10 min). Protein concentration of the lysate was determined using the DC Assay (BioRad) according to the manufacturer's protocol and 50 µg total lysate was used in SDS-Gel-Electrophoresis (15 % SDS-PAGE, Tris-Glycine). Proteins were transferred using semi-dry blotting (BioRad TransBlot® Turbo™ Transfer System) onto nitrocellulose membranes. The membrane was blocked with 1X TBST (5 % skim Milk powder) and washed 3x with TBST (50 mM Tris pH 7.5, 150 mM NaCl, 0.1% Tween 20). First and secondary antibodies were used at recommended dilutions in TBST with milk and washed 3x between and after incubations (1 h, 20 °C, rocking shaker). The blots were read out using Pierce™ ECL Western Blotting-Substrate (ThermoFisher).

### Lysate stability

Peptide stability was measured in HEK293T lysates. Cell pellets were stored at -80 °C, resuspended in 1x PBS and lysed by 3 repeats of freezing in liquid $N_2$ and thawing at 37 °C. After centrifugation (16873 x *g*, 10 min) the supernatant was used for the stability experiments. Time course experiments were performed by incubating 140 µl of a mix of 1 mM peptide and 1 mg/ml lysate at 37 °C. 10 µl samples were taken and mixed immediately in equal volume with ice cold ethylparaben in MeOH (0.05 mg/ml). The samples were chilled on ice for 15 min and insoluble material was removed by centrifugation (16873 x *g*, 4 °C, 10 min). The supernatant was analysed by LCMS with a gradient of $H_2O$ (0.1% TFA) and ACN (0.1% TFA) from 5 to 95 % over 20 min. The area under the curve of the peaks was integrated and normalized to the ethylparaben standard.

### NanoClick cell permeability assay

The assay was perfomed with HEK239T cells like reported by Peier et al..[7] HEK293T cells were diluted to reach a concentration of 200000 cells/ml in 10 ml and transfected with 15 µl FuGENE® transfection reagent (Promega) using 0.5 µg BRET plasmid (NanoBRET™ positive control vector; Promega N1581) and 4.5 µg carrier DNA (Promega N1581) mixed with 480 µl OptiMEM (no phenol red, Gibco). From this transfected mix, 100 µl were seeded into 96-well plates (Greiner white transparent bottom, **655094**) and were cultivated for 24 h. Then, the medium was replaced with 90 µl assay buffer (OptiMem without phenol red + 1 % FBS) and DIBAC-CA was added to reach a final concentration of 3 µM. After 1h of treatment the cells were washed twice with HBSS (with Mg and Ca), and the medium was replaced with 80 µl assay buffer. Directly, the cells were treated with titrations of azido-peptides (**P-6-Az**, **P-6-Az**, **R8-Az** and **ONEG-Az**) and incubated for 20 h. On the next day, the NB618AZ dye (Promega) was added to a final concentration of 10 µM and the cells were treated for 1 h. Then, 50 µl of 3X Intracellular TE Nano-Glo® Substrate/Inhibitor mix (Promega N2162) was added, and luminescence was read out using a TECAN Spark plate reader (415 – 430 nm and 595 – 635 nm). BRET ratios were calculated with background correction according to equation (1) as described in the Promega® protocol for the use of the Intracellular TE Nano-Glo® Substrate/Inhibitor.[8]

$$BRET\ ratio = \left(\frac{Acceptor_{Sample}}{Donor_{Sample}} - \frac{Acceptor_{NoTracerControl}}{Donor_{NoTracerControl}}\right) \cdot 1000 \qquad (1)$$

# 2. Synthetic methods

### Reagents

All solvents and reagents were obtained from commercial sources and used without any further purification unless stated otherwise. Solid phase peptide synthesis was performed manually in 20 ml polypropylene syringe reactors obtained from MultiSynTech GmbH and equipped with a Teflon two-way valve. Suspensions of peptidyl resins were agitated using a shaker at room temperature. Alternatively, the polypropylene fritted syringe reactors were mounted on a Vac-Man® Laboratory Vacuum Manifold equipped with polypropylene three-way valves for agitation with argon.

Intermediate evaluation during solid phase peptide synthesis was performed by cleaving a small amount of resin followed by analysis using either an Agilent 1200 HPLC equipped with an analytical EC HPLC column (NUCLEOSHELL RP 18, 5 µm, 50x4.6 mm) or an Agilent infinity UHPLC equipped with an Agilent ZORBAX Eclipse Plus column (2.1 mm x 50 mm, 1.8 µm Zorbax Eclipse C18 Rapid Resolution). Purity analysis of final peptides was performed using an Agilent infinity UHPLC equipped with an Agilent InfinityLab Poroshell 120 EC-C18, 2.1 x 150 mm, 2.7 µm, narrow bore LC column. An LTQ Orbitrap in tandem with an HPLC-System fitted with a 50 mm x 1 mm, 1.9 µm Hypersyl GOLD column was used to record HRMS data using electrospray ionization.

**Synthesis of Peptides**

**Linear Peptide Synthesis**

Peptide synthesis was performed on Rink-Amide-AM resin (1% DVB; 100-200 mesh,Carbolution Chemicals GmbH) as solid support typically on a 50-100 µmole scale. The resin was initially swollen using DCM (1 min × 1) and then washed twice with DMF (2 × 30s). Fmoc-deprotection was performed using 1:4 Piperidine in DMF (1 × 5 min; 1 × 10 min) and washed with DMF (4 × 30s). The first Fmoc-Xaa-OH (5.0 eq.) was dissolved in a freshly prepared solution of PyBOP (5.0 eq.) and DIPEA (10.0 eq.) and was added to the Fmoc-deprotected resin and shaken for 45 min at room temperature. The resin was then washed with DMF (3x), DCM (3x) and diethyl ether (3x) and dried under high vacuum. Afterwards, the loading efficiency of the resin was checked by measuring the UV-absorption at 300 nm of a small quantity of resin deprotected using the piperidine solution. A suitable amount of resin was than swollen again as before and extended using Fmoc protected amino acid (4 eq), PyBOP (4 eq) and DIPEA (8 eq) for 45 min - 1 h followed by washing with DMF (3x), DCM (3x) and DMF (3x). For the coupling of unnatural amino acid building blocks ($R_5$, $S_5$ and $B_5$), Fmoc-Xaa-OH (2.0 eq.) was dissolved in DMF in the presence of PyBOP (2.0 eq.), Oxyma (1.0 eq.) and DIPEA (4.0 eq.), added to the resin and shaken for overnight at room temperature. If necessary, amino acids following the non-natural amino acids amino acids used for stapling were coupled twice.

**Acetylation**

The Fmoc-deprotected peptidyl resin was suspended in DMF followed by the addition of $Ac_2O$ (10 eq) and DIPEA (10 eq). The suspension was shaken for 30 min and washed with DMF (2 × 30s).

**FITC Labelling**

The syringe reactor containing Fmoc-deprotected linear peptide on resin suspended in DMF was charged with Fmoc-O2Oc-OH (2 eq), PyBOP (2 eq) and DIPEA (4 eq) and shaken overnight. The resin was washed with DMF (2 × 30s), deprotected with 1:4 Piperidine in DMF (1 × 5 min; 1 × 10 min), washed with DMF (4 × 30s) and resuspended in DMF. The syringe reactor was then charged with FITC (1.5 eq) and DIPEA (4 eq) in DMF and shaken overnight. The peptidyl resin was then washed with DMF (4 × 30s), DCM (2 × 30s), and $Et_2O$ (1 × 1 min) and dried under vacuum.

**Ruthenium Olefin Metathesis**

Either one of the following protocols was used for the ring-closing metathesis reaction:

(i) A solution of Grubbs Catalyst® 1st Generation (0.1 equivalent) was prepared in degassed DCM (3 mL), then drawn into a 10 mL syringe reactor which contains the Fmoc-protected substrate on resin. The reactor was sealed and shaken at rt for 2 hours, followed by discharging the supernatant. The

substrate was treated by the same procedure with freshly prepared Grubbs Catalyst® 1st Generation solution for 3 more times. Afterwards, the resin was washed with DCM (2 x 30 s), dried under vacuum.

(ii) A solution of Hoveyda-Grubbs Catalyst® 2nd Generation (20 mol%) in DCE (2 mL) was drawn in a syringe reactor containing a suspension of Fmoc-protected peptide on resin. The reaction vessel was equipped with an open two-way valve and shaken for 2 h. The supernatant was then discharged and the syringe reactor was recharged with a fresh solution of catalyst and shaken. After 2 h, the peptidyl resin was washed with DMF (4 × 30s), DCM (2 × 30s), and $Et_2O$ (1 × 1 min) and dried under vacuum.

**Peptide cleavage**

Peptides were cleaved from the resin by treating it with a solution of TFA/TIPS/$H_2O$ (95:2.5:2.5) for 1 h followed by filtration of the resin. The filtrate was then added to ice cold $Et_2O$ and the crude peptide obtained by centrifugation. The pellet was resuspended in fresh ice cold $Et_2O$ followed by centrifugation. This procedure was repeated once more.

**Stapled Peptide Reduction**

If the double bond of the hydrocarbon staple was to be reduced, the substrate was first cleaved from the resin via the previously described cleavage protocol and then precipitated using cold diethyl ether followed by dissolution in MeOH/AcOH (3 mL, 1:1). The solution was transferred to a round-bottom flask which contained a stir bar and 10% Pd/C (30 mg), sealed with rubber stopper, and filled with argon. Next, the argon was exchanged with hydrogen and the mixture was stirred at rt for 2 h. After the reaction was complete (as monitored by LCMS), the reaction was filtered through a pad of celite. The residue was washed with MeOH, the filtrates combined, concentrated, and purified by preparative HPLC.

**Synthesis of azido-octa-arginine and azido-ONEG**

Peptides were synthesized using a PurePrep Chorus® Peptide synthesizer on Rink Amide MBHA resin (loading: 0.5mmol/g). Resin swelling was done in DCM at 25°C for 10 minutes. All couplings were performed using AA (5eq), HCTU (5eq) and DIPEA (10eq) in DMF at 50°C and 150 rpm for 10 minutes following by a capping cycle with $Ac_2O$ (10eq) and DIPEA (10eq) in DMF for 10 min. Deprotection steps were performed in two cycles of 1 and 5 min respectively with 20% Piperidine in DMF. In between steps the resin was washed 4 x with DMF. After the synthesis was completed, each resin was transferred into a manual peptide synthesis vessel and Fmoc-Lys($N_3$)-OH (2eq) was coupled using DIC (2eq) and Oxyma (2eq) overnight. After final Fmoc removal, the peptides were acetylated by treatment with $Ac_2O$ (10eq) and DIPEA (10eq).

**azido-octa-arginine** was treated with a TFA:TIS:DCM (95:2.5:2.5) solution for 2 hours to avoid possible remaining Pbf protecting groups. The TFA was removed under vacuum and the crude dissolved in $H_2O$/MeCN (1:1) and lyophilized before purification. Purification was carried out using a Büchi Pure C850 FlashPrep Chromatography system equipped with a NUCLEODUR C18 Gravity, 5 µm, 125x10 mm column, mobile phase $H_2O$:MeOH 0.01%TFA, from 0 to 30% MeOH in 30 min, and 30 to 100% until 40 min.

**azido-ONEG:** the peptide was deprotected and cleaved with a TFA:TIS:DCM (95:2.5:2.5) treatment for 1 hour followed by precipitation in cold $Et_2O$ and centrifugation. The supernatant was removed, and the pellet resuspended in cold $Et_2O$ followed by centrifugation. This procedure was repeated twice. The resulting pellet was dissolved in $H_2O$/MeCN (1:1) and lyophilized. Purification was carried out using a Büchi Pure C850 FlashPrep Chromatography system equipped with a NUCLEODUR C18 Gravity, 5 µm, 125x10 mm column, mobile phase $H_2O$:MeCN 0.01%TFA, from 0 to 50% MeCN in 30 min.

### Synthesis of dibenzoazacyclooctyne Chloroalkane (DIBAC-CA)

Synthesis was based on the protocol described by Peier et al.[7] DIBAC free acid (150.0 mg, 0.45 mmol) was dissolved in DMF and HATU (256.5 mg, 0.67 mmol), 2-(2-((6-chlorohexyl)oxy)ethoxy)ethan-1-amine (117 mg, 0.45 mmol) and N,N-diisopropylethylamine (234 uL, 1.35 mmol) were added at 0 °C and the mixture was stirred for 10 minutes. Then, the reaction mixture was allowed to reach room temperature and stirred for 4hs. The solvent was removed under reduced pressure, and the resulting residue was dissolved in $H_2O$:MeCN (50:50, 0.01%TFA) and purified using a Büchi Pure C850 FlashPrep Chromatography system equipped with a NUCLEODUR C18 Gravity, 5 µm, 125x10 mm column, mobile phase $H_2O$:MeCN 0.01%TFA, from 0 to 50%MeCN in 8 min, 50 to 80% at 20 min and 100% in 30 min. The product was then lyophilized and stored protected from light.Yield: 181 mg (79.0%) as a colorless oil.

**P-1**

Ac—N—Q—A—R—A—Q—A—A—L—Q—A—V—N—R—$NH_2$

**LRMS:** Exact mass (calculated) $[M+H]^+$: 776.43; Observed mass: 776.60

**Purification:** A Büchi Pure C-850 Flash/Prep system, using a C18 column (Macherey-Nagel, 5μM, 125 x 21 mm) was used for purification applying a linear gradient from 16% to 40% over 40 minutes at a flow rate of 20 ml/min, with a mobile phase composed of eluent A (99.9% v/v $H_2O$, 0.1% v/v TFA) and eluent B (99.9% v/v ACN and 0.1% v/v TFA).

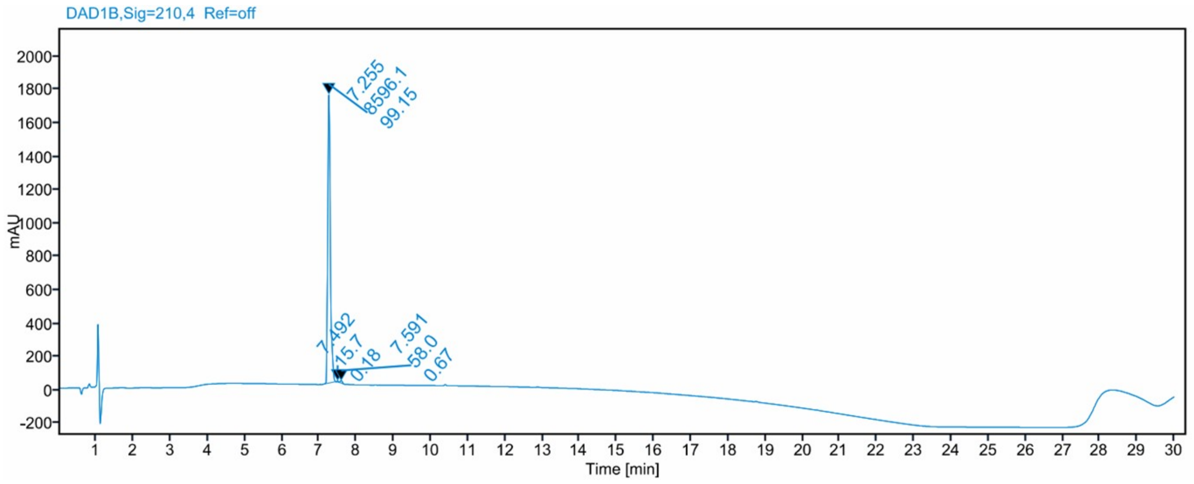

**P-2**

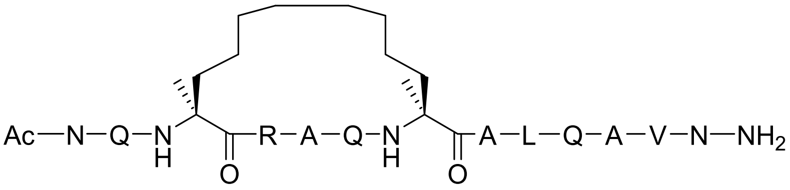

**HRMS:** Exact mass (calculated) $[M+H]^+$: 1505.8547; Observed mass: 1505.8480

**Purification:** A Büchi Pure C-850 Flash/Prep system, using a C18 column (Macherey-Nagel, 5µM, 125 x 21 mm) was used for purification applying a linear gradient from 16% to 40% over 40 minutes at a flow rate of 20 ml/min, with a mobile phase composed of eluent A (99.9% v/v $H_2O$, 0.1% v/v TFA) and eluent B (99.9% v/v ACN and 0.1% v/v TFA).

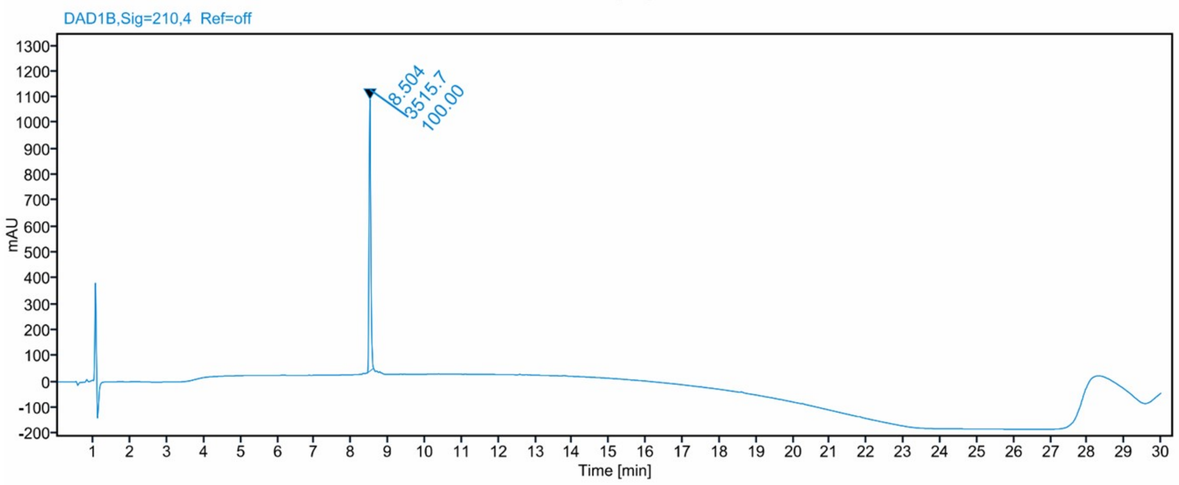

**P-3**

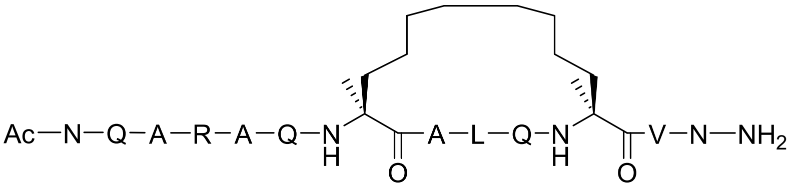


**HRMS:** Exact mass (calculated) $[M+H]^+$: 1505.8547; Observed mass: 1505.8581

**Purification:** A Büchi Pure C-850 Flash/Prep system, using a C18 column (Macherey-Nagel, 5µM, 125 x 21 mm) was used for purification applying a linear gradient from 16% to 40% over 40 minutes at a flow rate of 20 ml/min, with a mobile phase composed of eluent A (99.9% v/v $H_2O$, 0.1% v/v TFA) and eluent B (99.9% v/v ACN and 0.1% v/v TFA).

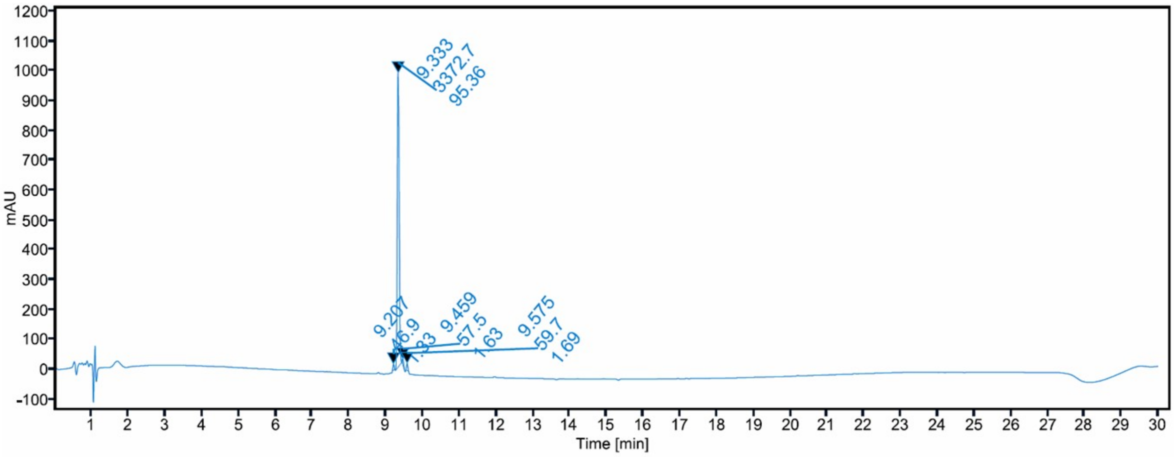

**P-4**

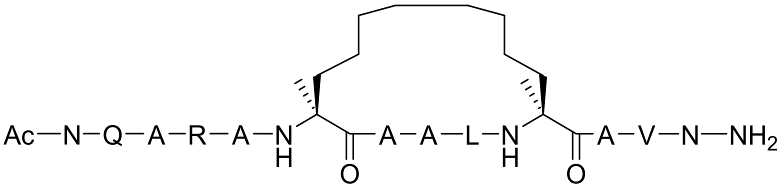


**HRMS:** Exact mass (calculated) $[M+H]^+$: 1391.8118; Observed mass: 1391.8148

**Purification:** A Büchi Pure C-850 Flash/Prep system, using a C18 column (Macherey-Nagel, 5µM, 125 x 21 mm) was used for purification applying a linear gradient from 16% to 40% over 40 minutes at a flow rate of 20 ml/min, with a mobile phase composed of eluent A (99.9% v/v $H_2O$, 0.1% v/v TFA) and eluent B (99.9% v/v ACN and 0.1% v/v TFA).

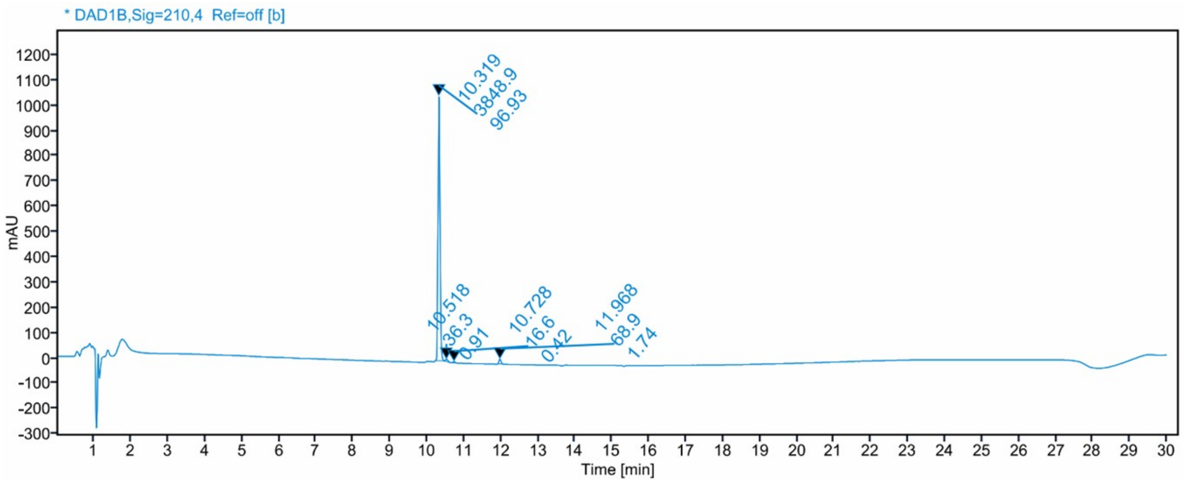

**P-5**

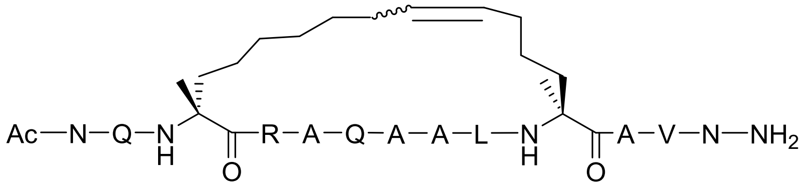


**HRMS:** Exact mass (calculated) $[M+H]^+$: 1488.8651; Observed mass: 1488.8670

**Purification:** An Agilent Infinity II LC-MS system, using a Zorbax 300SBC18 column (Agilent, Santa Clara, USA) was used for purification applying a linear gradient from 5% to 50% over 30 minutes at a flow rate of 6 ml/min, with a mobile phase composed of eluent A (99.9% v/v $H_2O$, 0.1% v/v TFA) and eluent B (99.9% v/v ACN and 0.1% v/v TFA).

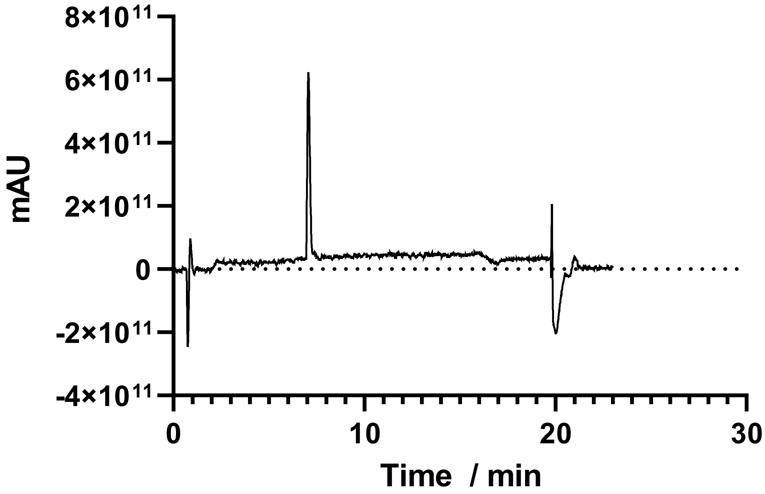

**P-6**

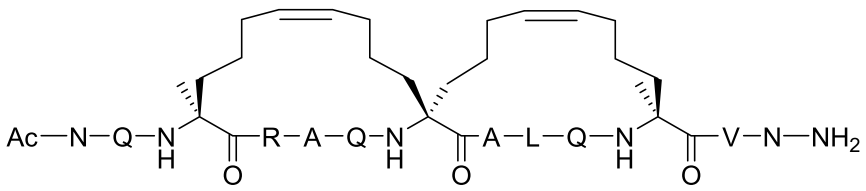


**HRMS:** Exact mass (calculated) $[M+H]^+$: 1597.9178; Observed mass: 1597.9205

**Purification:** A Büchi Pure C-850 Flash/Prep system, using a C18 column (Macherey-Nagel, 5µM, 125 x 21 mm) was used for purification applying a linear gradient from 0% to 95% over 60 minutes at a flow rate of 20 ml/min, with a mobile phase composed of eluent A (99.9% v/v $H_2O$, 0.1% v/v TFA) and eluent B (99.9% v/v ACN and 0.1% v/v TFA).

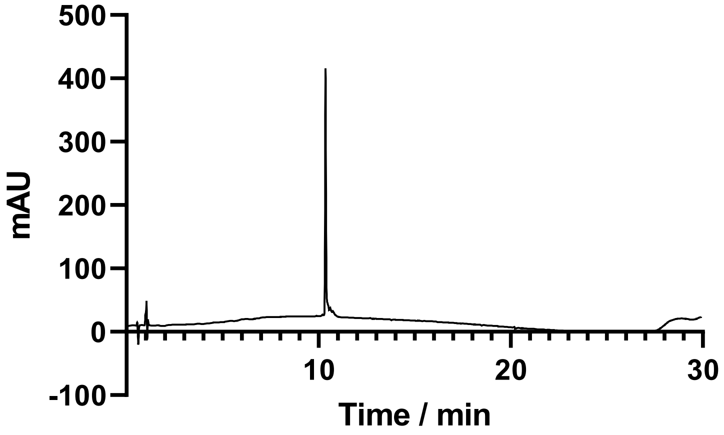

**P-7**

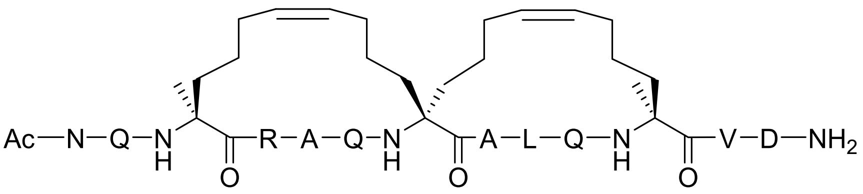


**HRMS:** Exact mass (calculated) $[M+H]^+$: 1598.9019; Observed mass: 1598.9013

**Purification:** A Büchi Pure C-850 Flash/Prep system, using a C18 column (Macherey-Nagel, 5µM, 125 x 21 mm) was used for purification applying a linear gradient from 0% to 95% over 60 minutes at a flow rate of 20 ml/min, with a mobile phase composed of eluent A (99.9% v/v $H_2O$, 0.1% v/v TFA) and eluent B (99.9% v/v ACN and 0.1% v/v TFA).

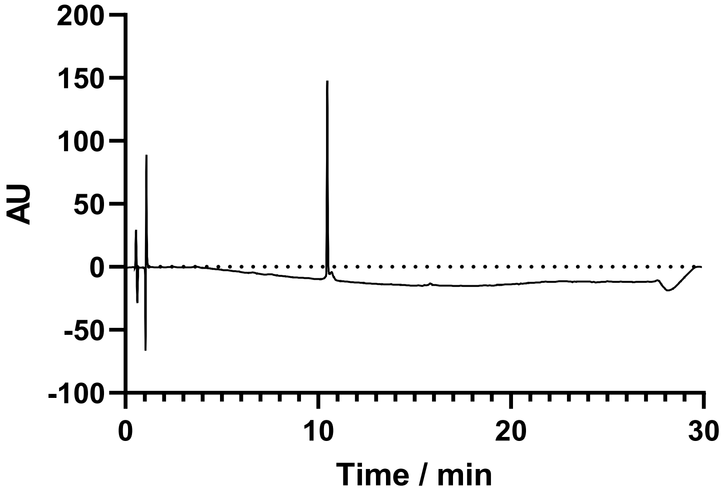

**P-8**

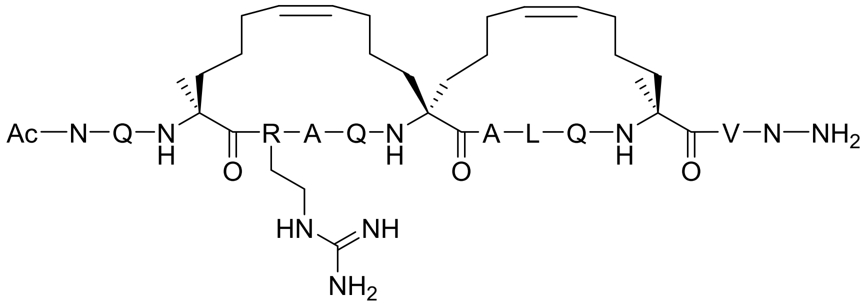


**HRMS:** Exact mass (calculated) $[M+H]^+$: 1583.9022; Observed mass: 1583.9056

**Purification:** A Büchi Pure C-850 Flash/Prep system, using a C18 column (Macherey-Nagel, 5µM, 125 x 21 mm) was used for purification applying a linear gradient from 0% to 95% over 60 minutes at a flow rate of 20 ml/min, with a mobile phase composed of eluent A (99.9% v/v $H_2O$, 0.1% v/v TFA) and eluent B (99.9% v/v ACN and 0.1% v/v TFA).

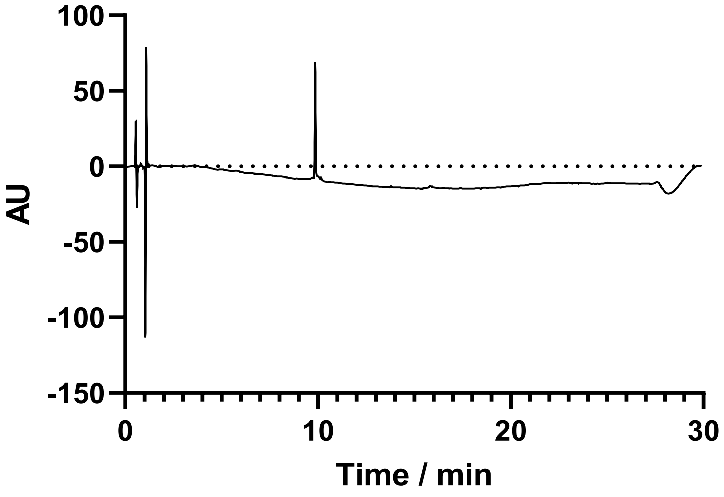

**P-9**

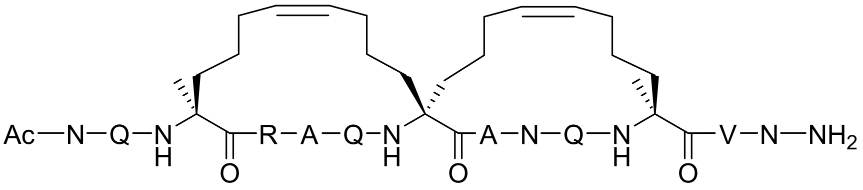


**HRMS:** Exact mass (calculated) $[M+H]^+$: 1598.8767; Observed mass: 1598.8805

**Purification:** A Büchi Pure C-850 Flash/Prep system, using a C18 column (Macherey-Nagel, 5µM, 125 x 21 mm) was used for purification applying a linear gradient from 0% to 95% over 60 minutes at a flow rate of 20 ml/min, with a mobile phase composed of eluent A (99.9% v/v $H_2O$, 0.1% v/v TFA) and eluent B (99.9% v/v ACN and 0.1% v/v TFA).

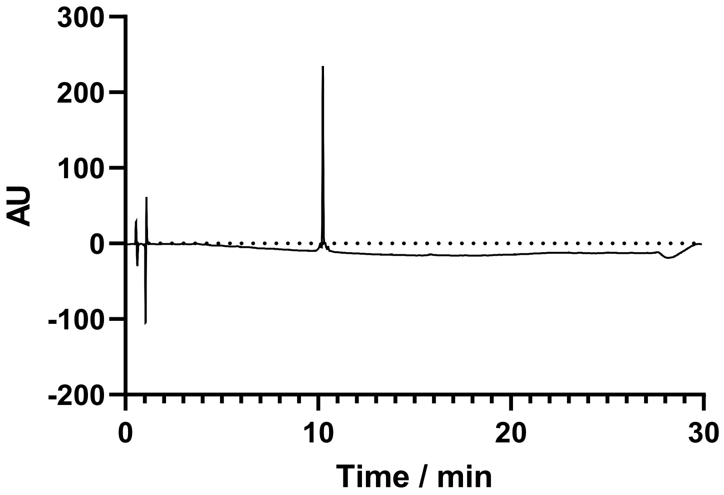

**P-10**

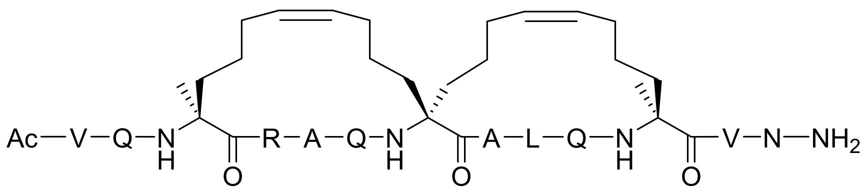


**HRMS:** Exact mass (calculated) $[M+2H]^{2+}$: 791.9756; Observed mass: 791.9787

**Purification:** A Büchi Pure C-850 Flash/Prep system, using a C18 column (Macherey-Nagel, 5µM, 125 x 21 mm) was used for purification applying a linear gradient from 0% to 95% over 60 minutes at a flow rate of 20 ml/min, with a mobile phase composed of eluent A (99.9% v/v $H_2O$, 0.1% v/v TFA) and eluent B (99.9% v/v ACN and 0.1% v/v TFA).

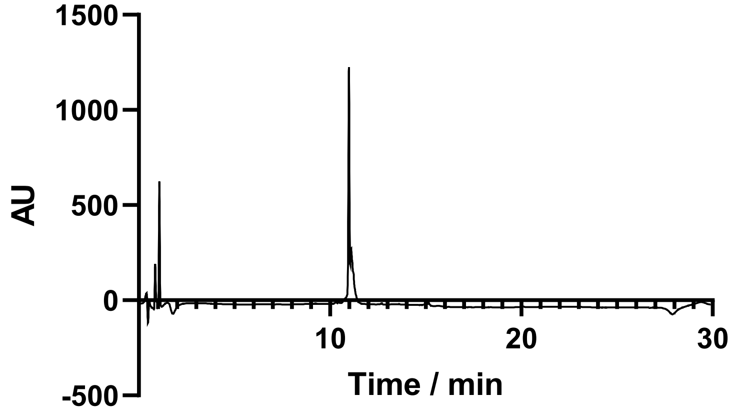

**P-6-Az:**

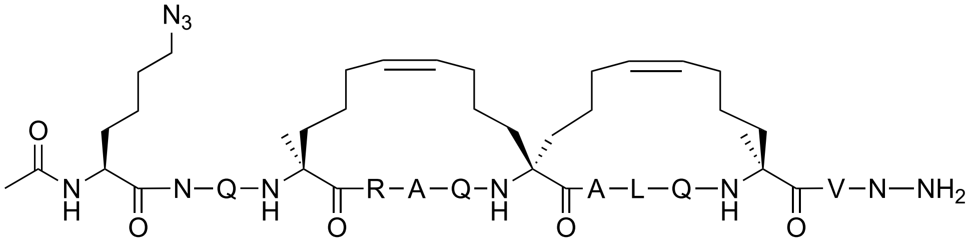


**HRMS:** Exact mass (calculated) $[M+2H]^{2+}$: 876.50501; Observed mass: 876.50697

**Purification:** A Büchi Pure C-850 Flash/Prep system, using a C18 column (Macherey-Nagel, 5µM, 125 x 21 mm) was used for purification applying a linear gradient from 0% to 95% over 60 minutes at a flow rate of 20 ml/min, with a mobile phase composed of eluent A (99.9% v/v $H_2O$, 0.1% v/v TFA) and eluent B (99.9% v/v ACN and 0.1% v/v TFA).

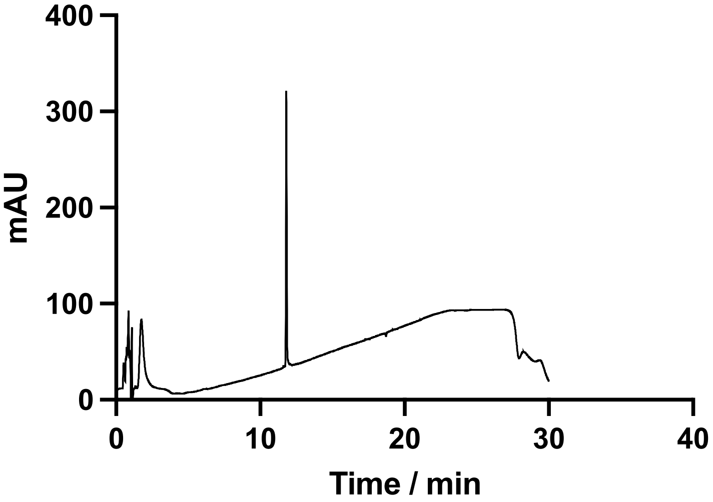

**P-6S-Az:**

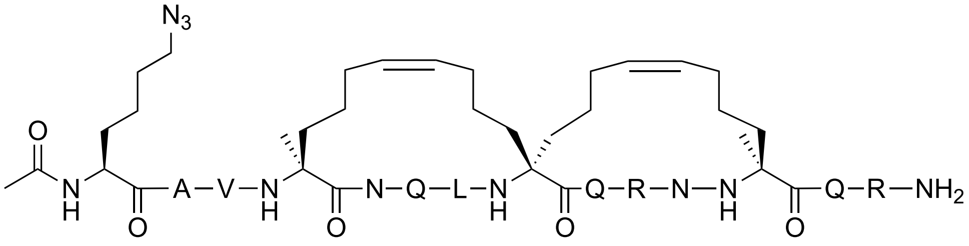


**HRMS:** Exact mass (calculated) $[M+2H]^{2+}$: 876.50501; Observed mass: 876.50711

**Purification:** A Büchi Pure C-850 Flash/Prep system, using a C18 column (Macherey-Nagel, 5µM, 125 x 21 mm) was used for purification applying a linear gradient from 0% to 95% over 60 minutes at a flow rate of 20 ml/min, with a mobile phase composed of eluent A (99.9% v/v $H_2O$, 0.1% v/v TFA) and eluent B (99.9% v/v ACN and 0.1% v/v TFA).

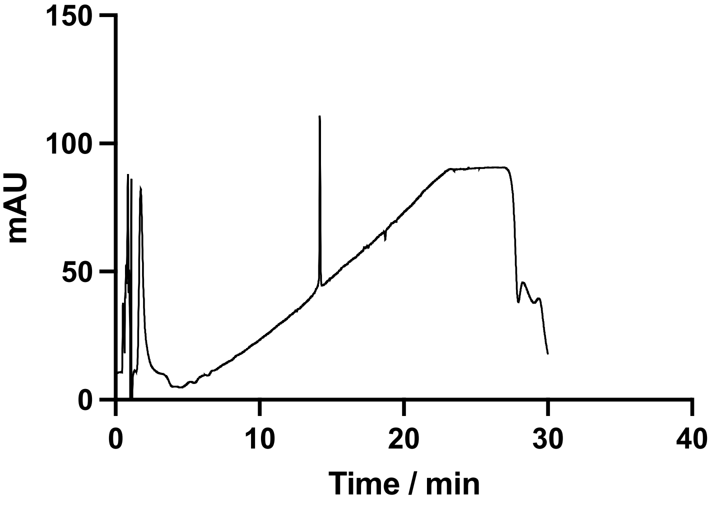

**P-6S:**

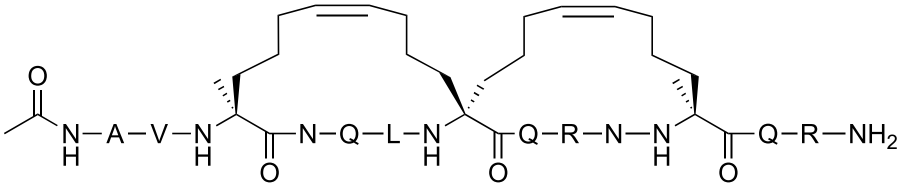


**HRMS:** Exact mass (calculated) $[M+2H]^{2+}$: 799.46228 ; Observed mass: 799.46410

**Purification:** A Büchi Pure C-850 Flash/Prep system, using a C18 column (Macherey-Nagel, 5μM, 125 x 21 mm) was used for purification applying a linear gradient from 0% to 95% over 60 minutes at a flow rate of 20 ml/min, with a mobile phase composed of eluent A (99.9% v/v $H_2O$, 0.1% v/v TFA) and eluent B (99.9% v/v ACN and 0.1% v/v TFA).

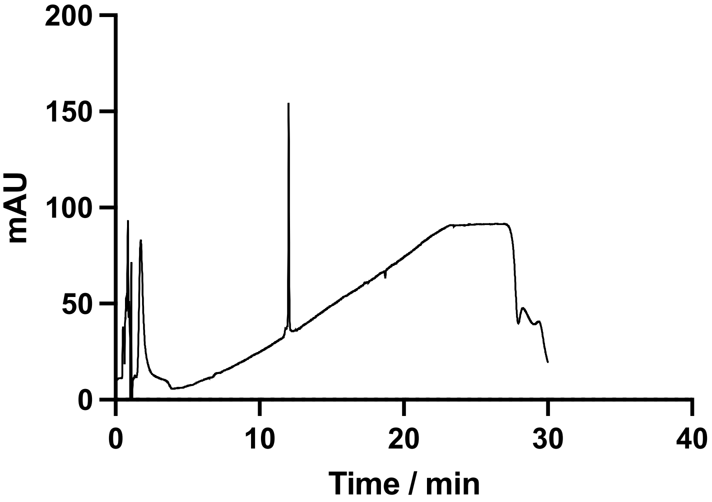

**P-6F:**

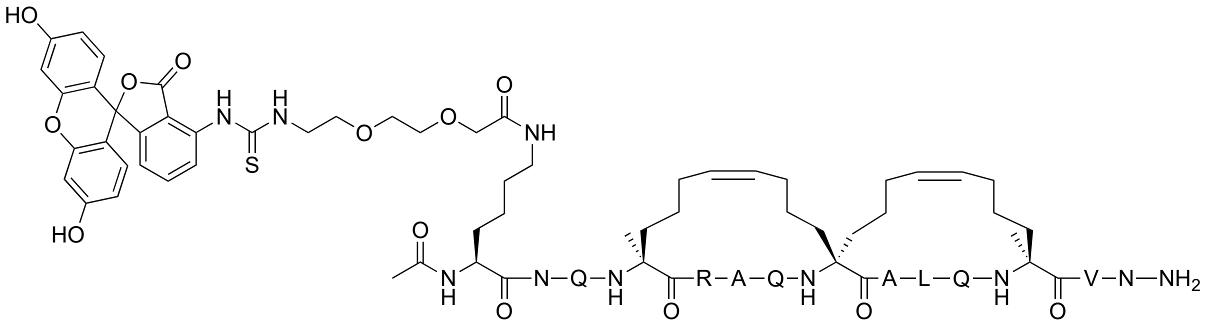


**HRMS:** Exact mass (calculated) $[M+2H]^{2+}$: 1130.56461; Observed mass: 1130.56743

**Purification:** A Büchi Pure C-850 Flash/Prep system, using a C18 column (Macherey-Nagel, 5μM, 125 x 21 mm) was used for purification applying a linear gradient from 0% to 95% over 60 minutes at a flow rate of 20 ml/min, with a mobile phase composed of eluent A (99.9% v/v $H_2O$, 0.1% v/v TFA) and eluent B (99.9% v/v ACN and 0.1% v/v TFA).

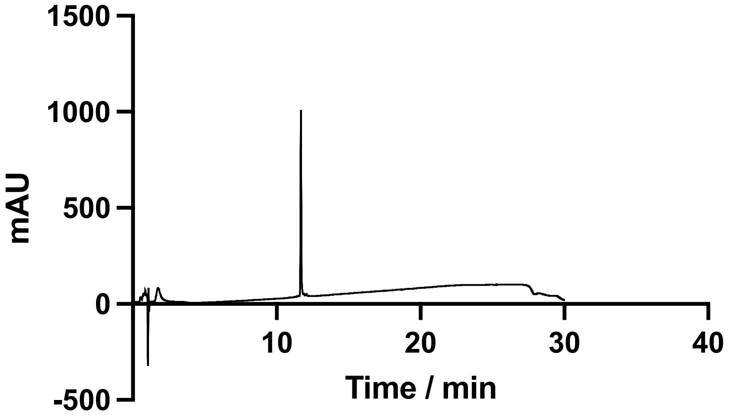

**DIBAC-CA:**

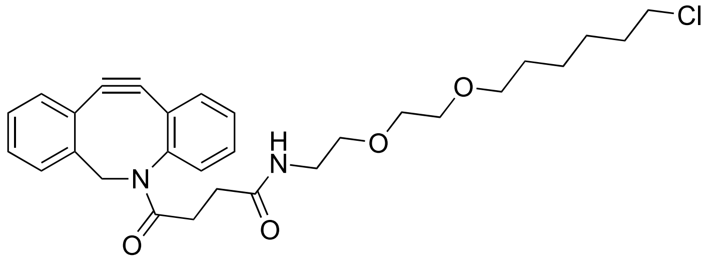


**LRMS:** Exact mass (calculated) $[M+H]^+$: 511.23; Observed mass $[M+H]^+$: 511.80

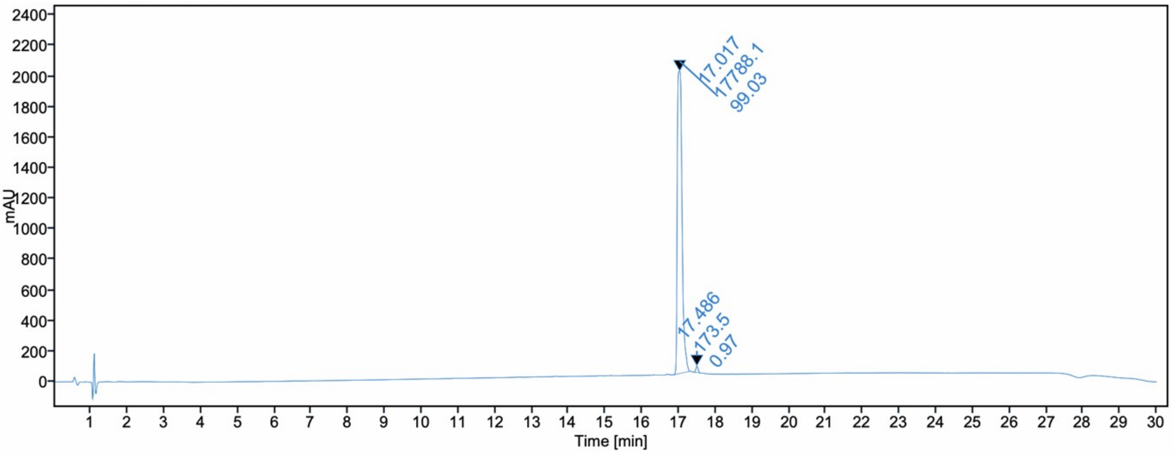

**azido-ONEG:**

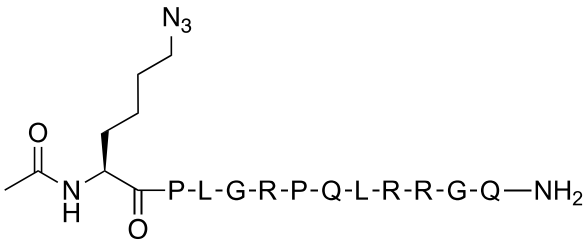


**LRMS**: Exact mass (calculated) $[M+2H]^{2+}$: 736.95, detected mass $[M+2H]^{2+}$: 737.00

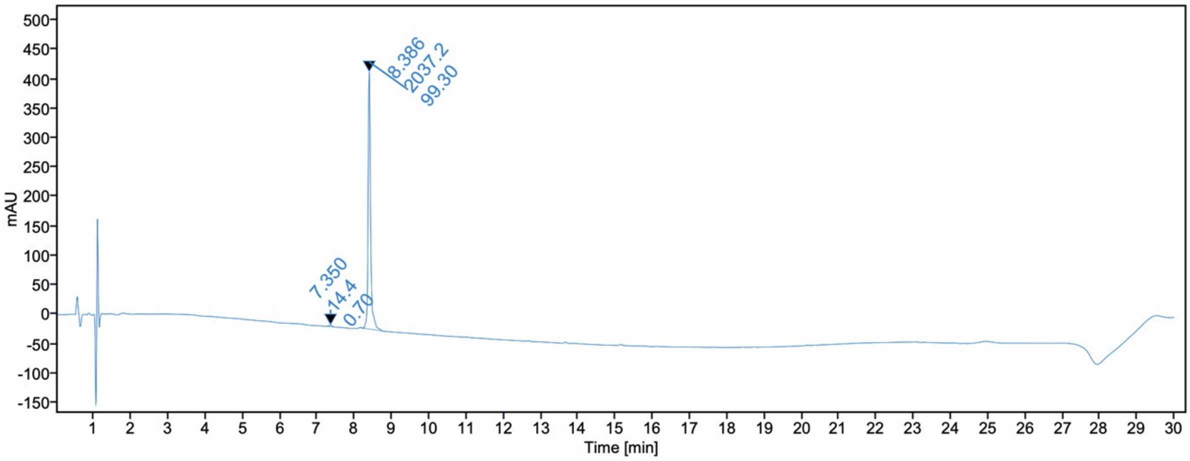

**Azido-octa-arginine:**

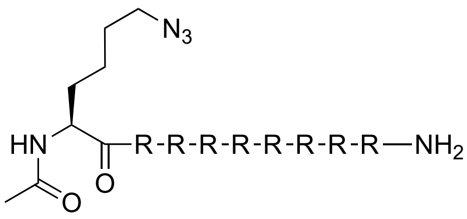


**LRMS:** Exact mass (calculated) $[M+3H]^{3+}$: 488.3, detected mass $[M+3H]^{3+}$: 488.6

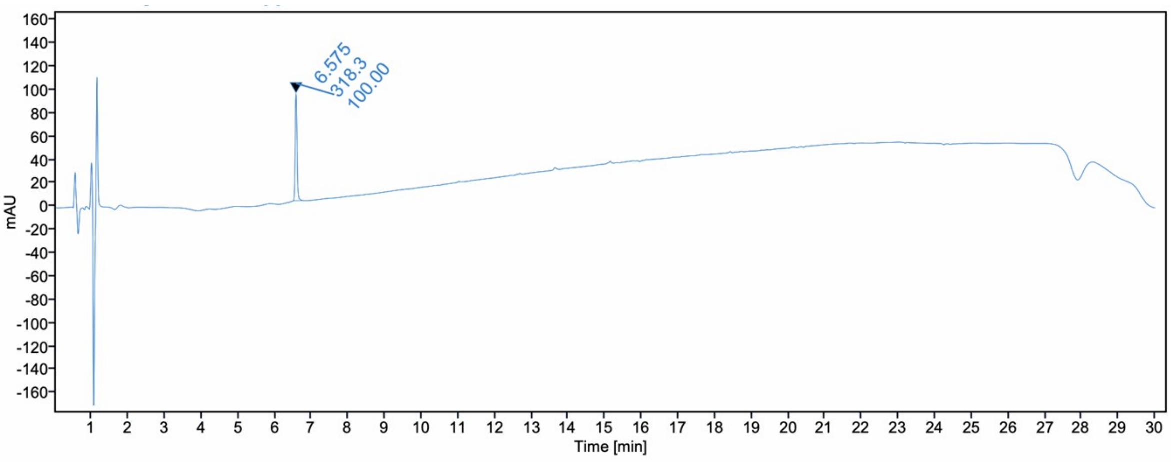

# 3. Figures

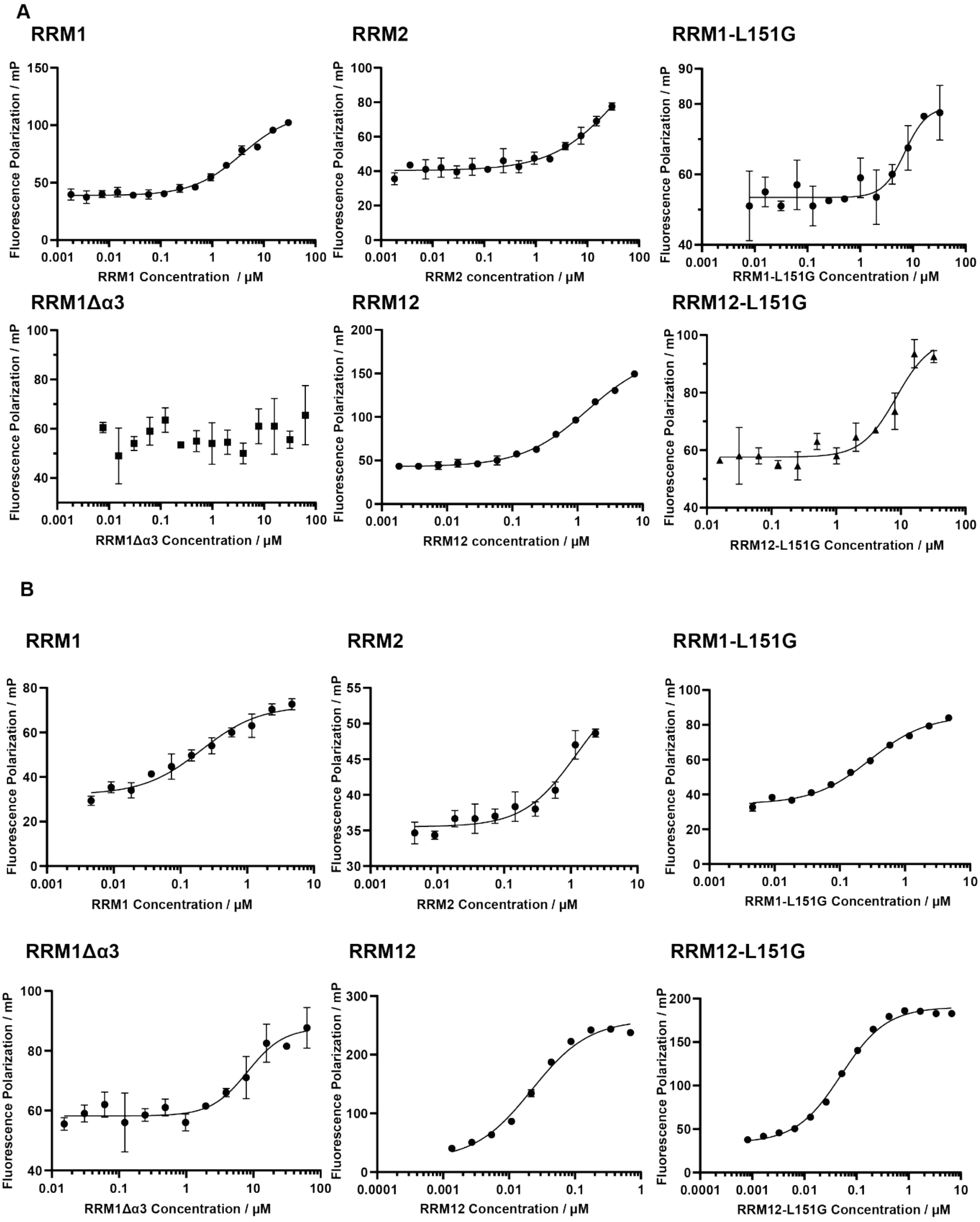


**Supplemental Figure 1:** Averaged fluorescence polarization curves of binding experiments of the PTBP1 constructs with **RNA-1** (**A**) and **RNA-2** (**B**).

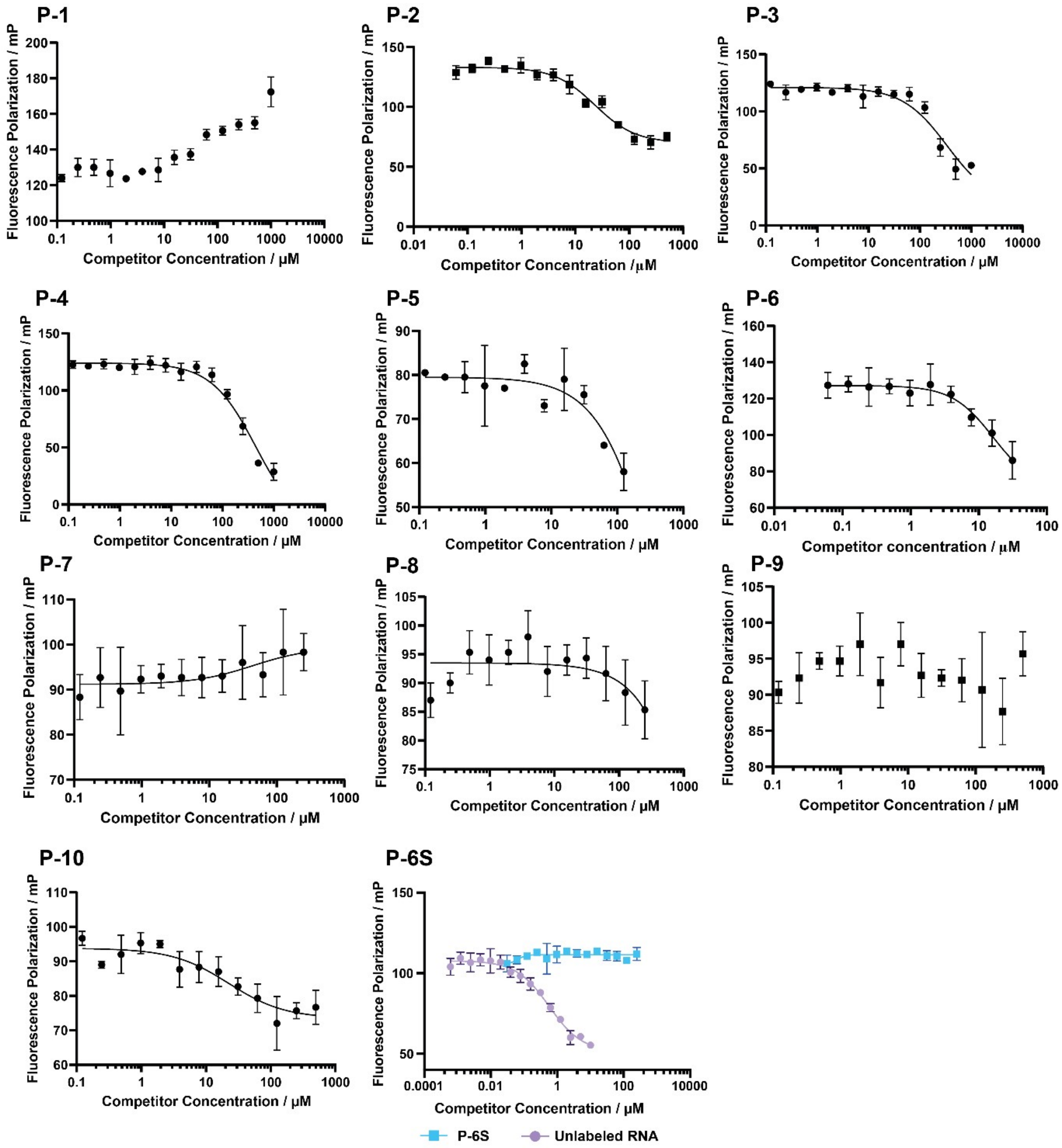


**Supplemental Figure 2**: Averaged fluorescence polarization curves of competitive experiments of PTBP1 RRM12, **RNA-2** with peptides **P-1** to **P-10,** and **P-6S**.

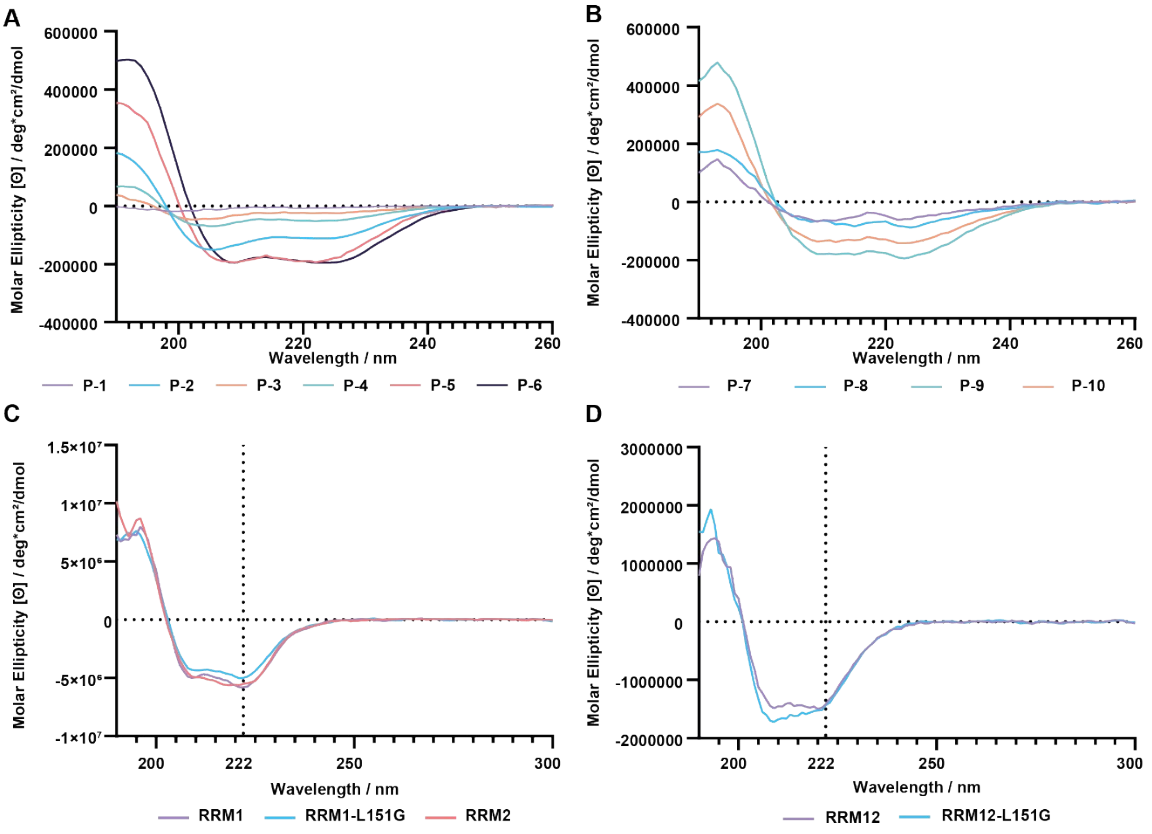


**Supplemental Figure 3:** Circular dichroism spectra of peptides **P-1** to **P-6** (**A**), **P-7** to **P-10** (**B**), single RRM constructs RRM1, RRM1-L151G and RRM2 (**C**), and tandem constructs RRM12 and RRM12-L151G (**D**).

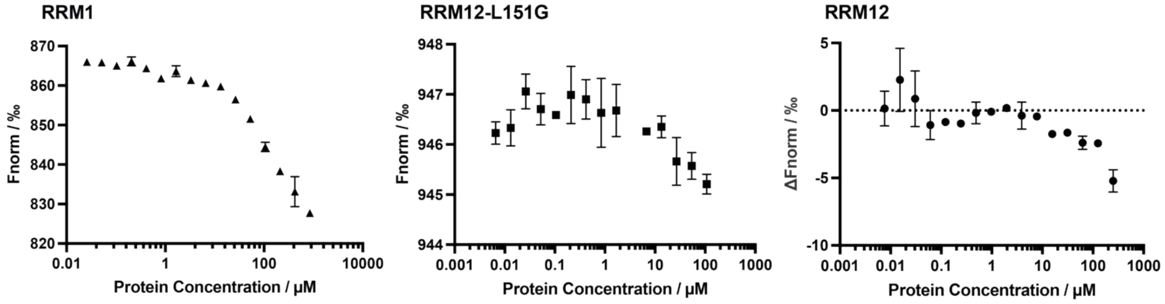


**Supplemental Figure 4:** Microscale thermophoresis assay of **P-6F** with RRM1, RRM12-L151G, and RRM12.

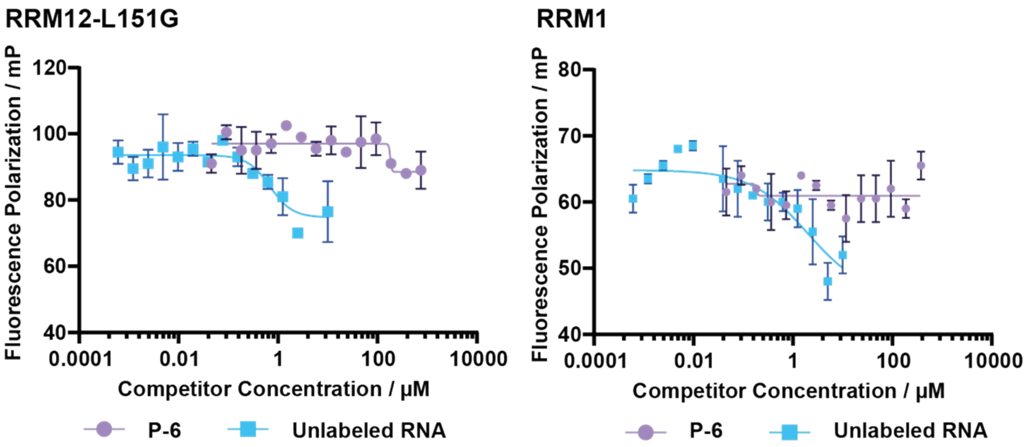


**Supplemental Figure 5:** Averaged fluorescence polarization curves of competitive experiments with **RNA-2**, RRM1 and RRM12-L151G with peptides **P-6** and the positive control of unlabeled **RNA-2**.

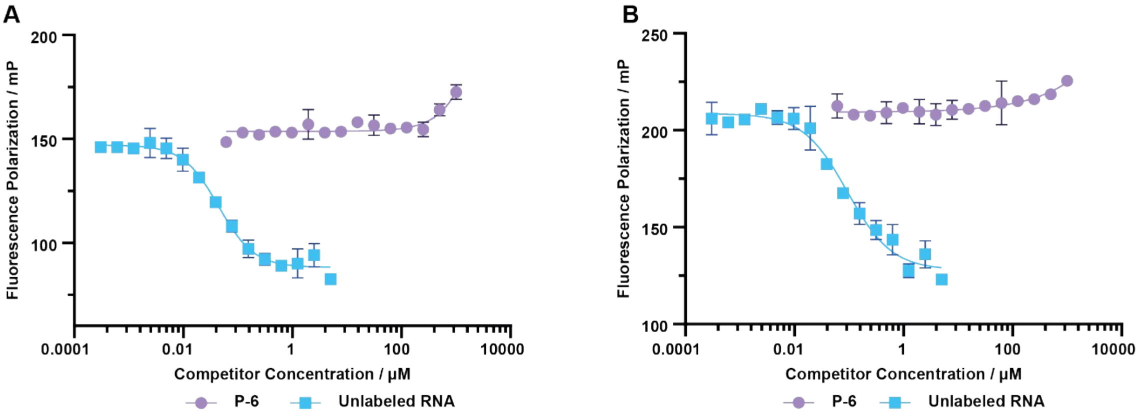


**Supplemental Figure 6:** Competitive fluorescence polarization experiments of **P-6** with hNRNPA2/B1 (**A**) and SRSF1 (**B**) with Cy5 labelled **RNA-AB** and **RNA-S** respectively. The unlabeled equivalents of the **RNA-AB** and **RNA-S** were used as positive controls.

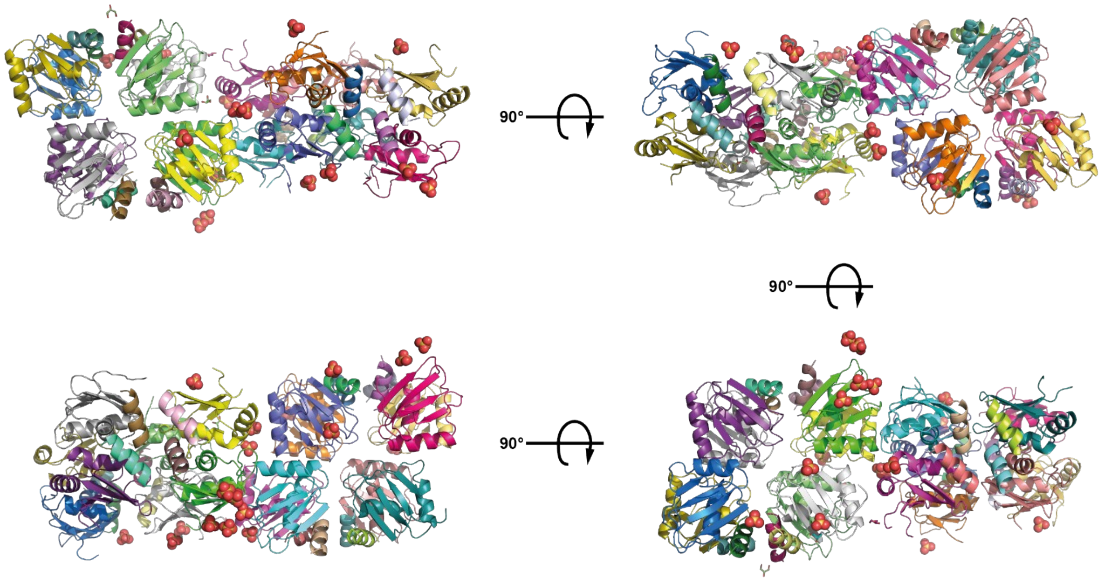


**Supplemental Figure 7:** Asymmetric unit of the crystal of RRM1 lacking the *C*-terminal α3 helix co-crystallized with **P-6**. The asymmetric unit contains 32 chains in total and is composed of 16 dimers of protein in complex with peptide.

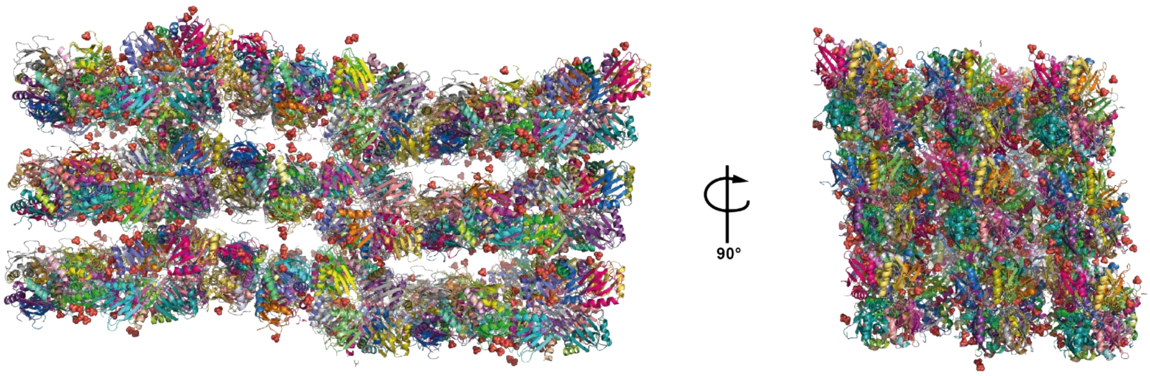


**Supplemental Figure 8:** Symmetry mates 50 Å around the ASU show a dense packing of the crystal without significant gaps.

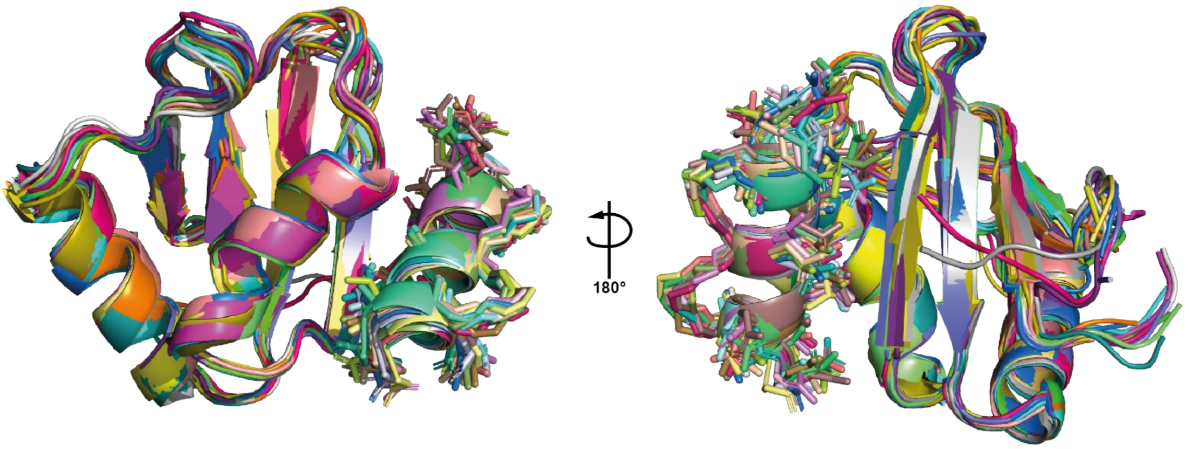


**Supplemental Figure 9:** Comparison of the 16 dimes in the ASU with stick representation of the ligands show a similar conformation and orientation of the monomers.

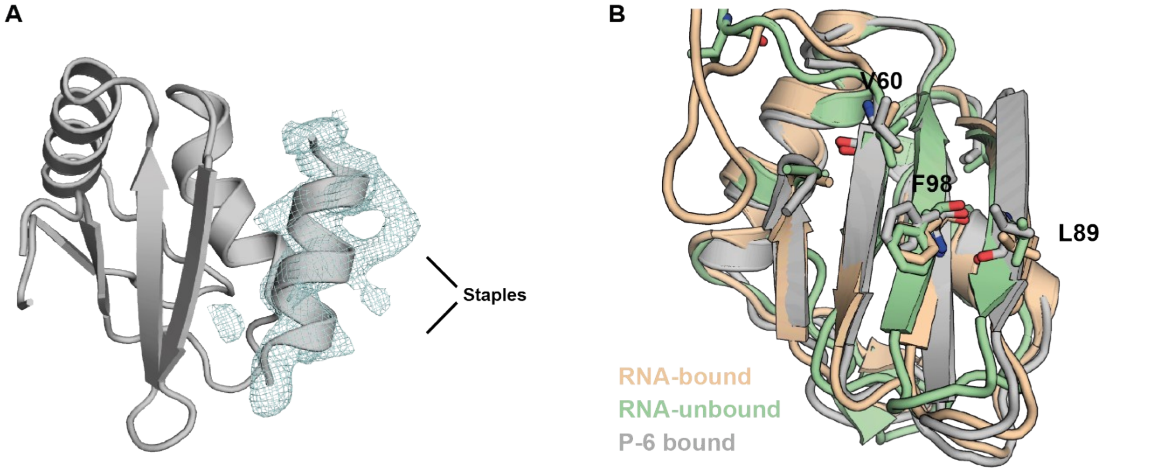


**Supplemental Figure 10: A** 2Fo-Fc map showing the helical density of **P-6** with the additional density of the staples. **B** Comparison of reported NMR structures of RNA bound RRM1 (PDB 2n3o), RNA-unbound RRM1 (PDB 1sjq) and **P-6** bound RRM1. Three key residues V60, F98 and L89 which organize the *C*-terminal loops orientation on the RNA binding site have the same orientation in all structures.

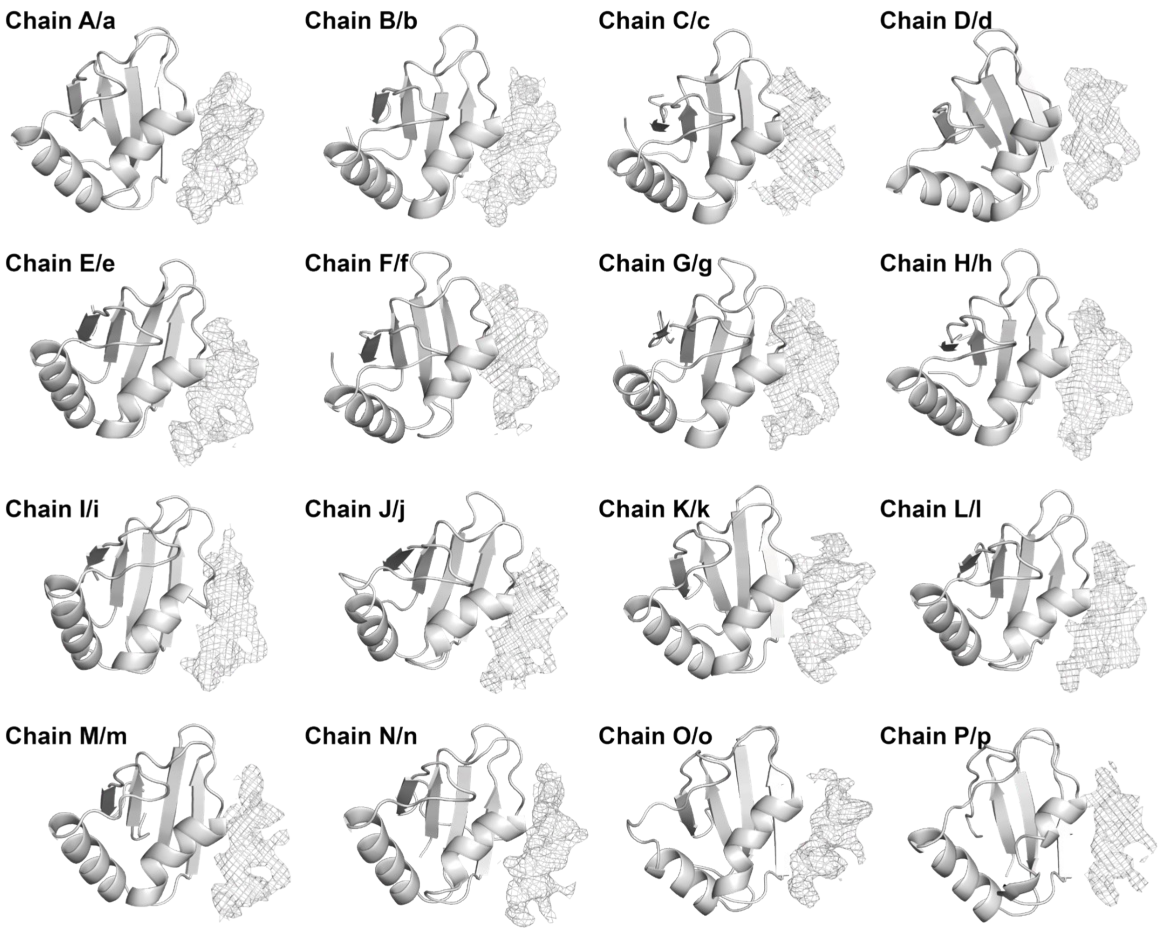


**Supplemental Figure 11:** 2Fo-Fc maps of the helical peptide omitting **P-6** for all 16 RRM1-**P-6** dimers in the unit cell of the crystal.

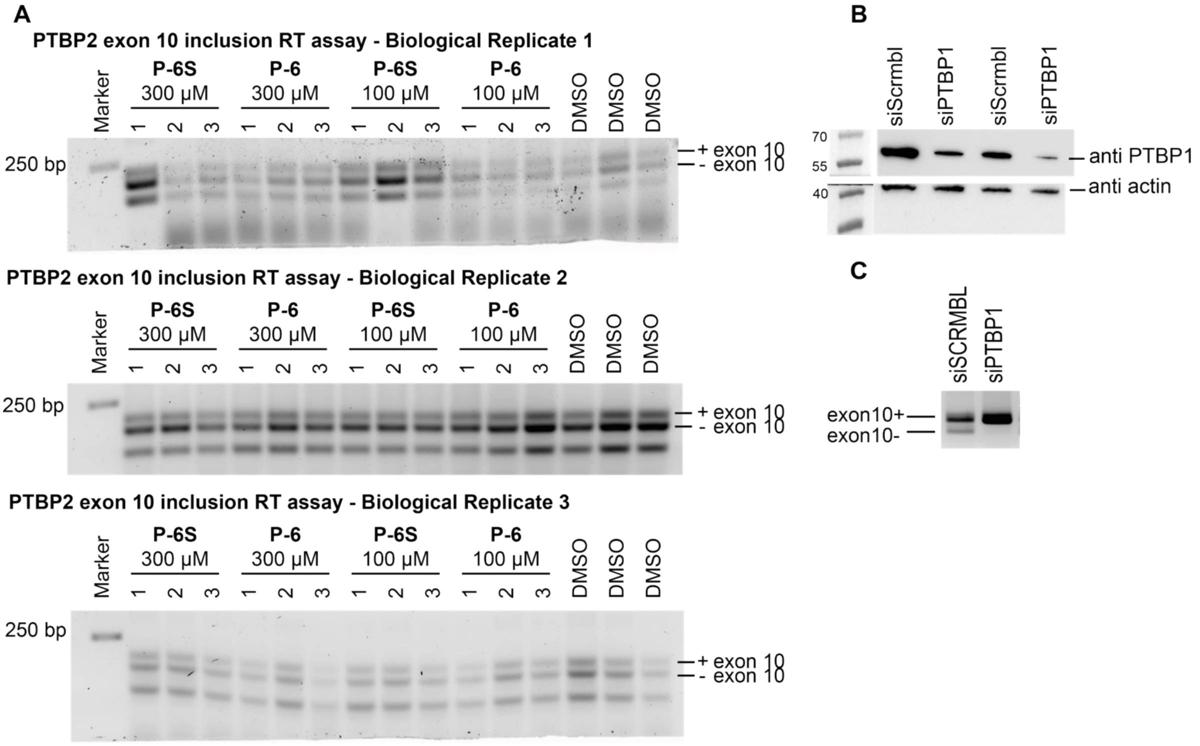


**Supplemental Figure 12**: (**A**) RT analysis of PTBP2 exon 10 inclusion in three biological replicates. (**B**) Western blot analysis shown in two replicates after knockdown using siPTBP1 which lowered the expression level of PTBP1 in HEK293T cells. (**C**) Knockdown of PTBP1 with siPTBP1 increased exon10 inclusion of the PTBP2 mRNA.

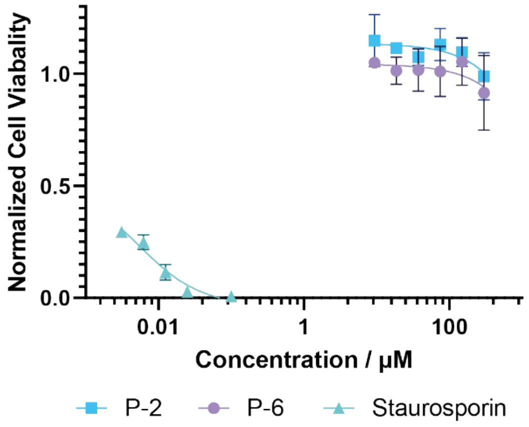


**Supplemental Figure 13:** Effect of **P-2** and **P-6** on cell viability measured with CellTiterGlo 2.0. Measured in two biological duplicates of technical triplicates.

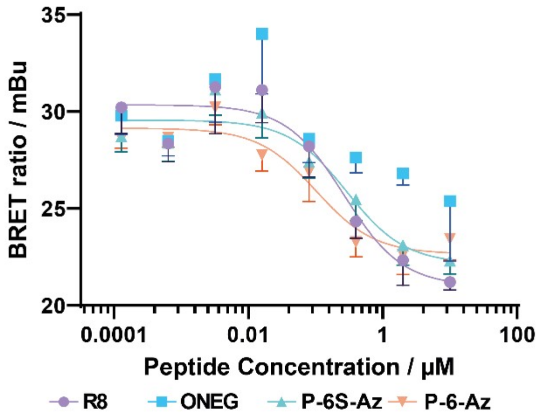


**Supplemental Figure 14:** Averaged BRET ratio for peptides **R8** (positive control) **ONEG** (negative control), **P-6S-Az**, and **P-6-Az** without background correction from three biological replicates.

# 4. Tables

**Table 1:** Data collection and refinement statistics.

| | RRM1Δ3-P6 |
|---|---|
| **Data collection** | |
| Space group | P 21 21 2 |
| Cell dimensions | |
| *a*, *b*, *c* (Å) | 244.2, 76.63, 94.13 |
| a, b, g (°) | 90, 90 ,90 |
| Resolution (Å) | 43.92-2.9 (3.004 – 2.9) |
| $R_{merge}$ | 0.0653 (1.497) |
| *I* / s*I* | 14.58 (1.17) |
| Completeness (%) | 99.31 (99.34) |
| Redundancy (Multiplicity) | 4.6 (4.8) |
| CC1/2 | 0.999 (0.554) |
| **Refinement** | |
| Resolution (Å) | 2.9 |
| No. Reflections | 39815 (3904) |
| $R_{work}$ / $R_{free}$ | 0.2431 / 0.3403 |
| No. Atoms | 11682 |
| Protein | 11264 |
| Ligand/ion | 418 |
| Water | 0 |
| ***B*-factors** | |
| Protein | 103.52 |
| Ligand/ion | 93.53 |
| **R.m.s. deviations** | |
| Bond lengths (Å) | 0.012 |
| Bond angles (°) | 1.39 |
| Ramachandran favored (%) | 90.092 |
| Ramachandran allowed (%) | 8.27 |
| Ramachandran outliers (%) | 0.80 |
| Rotamer outliers (%) | 5.88 |
| Clashscore | 25.86 |

**Table 2:** Comparison of the 16 monomers of the ASU by RMSD values with each other and to an RNA-bound structure (PDB 2n3o) and RNA-unbound structure (PDB 1sjq).

| Chain | RMSD to chain A / Å | RMSD to RNA bound / Å | RMSD to RNA unbound / Å |
|---|---|---|---|
| **A** | - | 1.329 | 1.595 |
| **B** | 0.45 | 1.45 | 1.58 |
| **C** | 0.687 | **1.144** | **1.482** |
| **D** | 0.718 | 1.48 | 1.643 |
| **E** | 0.728 | 1.306 | 1.549 |
| **F** | 0.691 | 1.382 | 1.612 |
| **G** | 0.712 | 1.431 | 1.534 |
| **H** | 0.697 | 1.513 | 1.659 |
| **I** | 0.723 | 1.357 | 1.587 |
| **J** | 0.776 | 1.42 | 1.73 |
| **K** | 0.596 | 1.418 | 1.66 |
| **L** | 0.684 | 1.399 | 1.536 |
| **M** | 0.894 | 1.423 | 1.563 |
| **N** | 0.725 | 1.464 | 1.734 |
| **O** | 0.886 | 1.564 | 1.554 |
| **P** | 0.882 | 1.523 | 1.678 |

# 5. Biologicals

**Used Oligonucleotides, Antibodies and siRNAs**

| Primer Pair | Primer 1 | Primer 2 | Purpose |
|---|---|---|---|
| RRM12 | TTTCAGGGCCATATGGGATCCCCCTCTAGAGTGATCCACATCCG | CTCAAGCTTGAATTCCTCGAGTTAGACGTTGAGGCTGGTGAGCT | Cloning |
| RRM12_a3_L151G | ttcaccgcctgcccggccgcctgggc | gcccaggcggccgggcaggcggtgaa | Mutagenesis |
| RRM1_a3_L151G | ttcaccgcctgcccggccgcctgggc | gcccaggcggccgggcaggcggtgaa | Mutagenesis |
| RRM1 | TTTCAGGGCCATATGGGATCCgcggaaatgacagcaagaagttcaaagg | CTCAAGCTTGAATTCCTCGAaaTTAggccaggttccccg | Cloning |
| RRM2 | TTTCAGGGCCATATGGGATCCatggccgggcagagc | CTCAAGCTTGAATTCCTCGAGTTAgtccccggaaggcagg | Cloning |
| PTBP2 | AGCTGCTGCTGGCCGAGTG | GATTGGTTTCCATCAGCCATCTG | RT-PCR |
| PTBP3 | CTTCCTCTGCTCGCGGTTAG | AGGTCCGTTAATGATGCCAGA | RT-PCR |

**FP Probes**

| Name | Sequence | Vendor |
|---|---|---|
| RNA-1 | FAM-GUCUUAA | Sigma Aldrich |
| RNA-2 | FAM-AUUUUUCCAUCUUUGUAUC | IDT |
| Unlabeled Probe | AUUUUUCCAUCUUUGUAUC | IDT |
| RNA-S | Cy5-AGAAGAACAGAAGAACAGAAGAAC | Sigma Aldrich |
| RNA-AB | AAGGACUAGC-Cy5 | Sigma Aldrich |

**Antibodies**

| Target | Vendor | Catalog # |
|---|---|---|
| Mouse anti-PTBP1 | Santa Cruz Biotechnology, Inc. | sc-56701 |
| Mouse anti-actin | Santa Cruz Biotechnology, Inc. | sc-8432 |
| Goat anti-mouse HRP-conjugate | Abcam PLC | ab205719 |

**siRNAs**

| Target | Vendor | Catalog # |
|---|---|---|
| PTBP1 | Horizon Discovery Group plc | L-003528-00-0005 |
| Control siRNA | Horizon Discovery Group plc | D-001810-01-05 |

## References


[1] M. Z. Lie, E. Stephen J., in *Gene Synthesis* (Ed.: J. pdf Peccoud), Humana Press, Totowa, NJ, **2012**, pp. 51–59.

[2] J. Quan, J. Tian, *Nature Protocols* **2011**, *6*, 242–251.

[3] W. Kabsch, *Acta Crystallographica Section D Biological Crystallography* **2010**, *66*, 125–132.

[4] A. J. McCoy, R. W. Grosse-Kunstleve, P. D. Adams, M. D. Winn, L. C. Storoni, R. J. Read, *Journal of Applied Crystallography* **2007**, *40*, 658–674.

[5] D. Liebschner, P. V. Afonine, M. L. Baker, G. Bunkóczi, V. B. Chen, T. I. Croll, B. Hintze, L.-W. Hung, S. Jain, A. J. McCoy, N. W. Moriarty, R. D. Oeffner, B. K. Poon, M. G. Prisant, R. J. Read, J. S. Richardson, D. C. Richardson, M. D. Sammito, O. V. Sobolev, D. H. Stockwell, T. C. Terwilliger, A. G. Urzhumtsev, L. L. Videau, C. J. Williams, P. D. Adams, *Acta Crystallographica Section D Structural Biology* **2019**, *75*, 861–877.

[6] P. Emsley, B. Lohkamp, W. G. Scott, K. Cowtan, *Acta Crystallographica Section D: Biological Crystallography* **2010**, *66*, 486–501.

[7] A. Peier, L. Ge, N. Boyer, J. Frost, R. Duggal, K. Biswas, S. Edmondson, J. D. Hermes, L. Yan, C. Zimprich, A. Sadruddin, H. Y. Kristal Kaan, A. Chandramohan, C. J. Brown, D. Thean, X. E. Lee, T. Y. Yuen, F. J. Ferrer-Gago, C. W. Johannes, D. P. Lane, B. Sherborne, C. Corona, M. B. Robers, T. K. Sawyer, A. W. Partridge, *ACS Chem. Biol.* **2021**, *16*, 293–309.

[8] https://www.promega.de/-/media/files/resources/protocols/technical-manuals/500/intracellular-te-nanoglo-substrate-inhibitor-and-nanoglo-vivazine-inhibitor-protocol.pdf?rev=128d75627a404a8eb0a1cbd7d9b92c1b&sc_lang=en
